# Soil redox status governs within-field spatial variation in microbial arsenic methylation and rice straighthead disease

**DOI:** 10.1093/ismejo/wrae057

**Published:** 2024-04-02

**Authors:** A-Xiang Gao, Chuan Chen, Zi-Yu Gao, Zhi-Qiang Zhai, Peng Wang, Si-Yu Zhang, Fang-Jie Zhao

**Affiliations:** State Key Laboratory of Crop Genetics and Germplasm Enhancement and Utilization, Center of Agricultural Health, Academy for Advanced Interdisciplinary, Jiangsu Provincial Key Laboratory for Organic Solid Waste Utilization, Jiangsu Collaborative Innovation Center for Solid Organic Waste Resource Utilization, College of Resources and Environmental Sciences, Nanjing Agricultural University, NO. 1 Weigang, Xuanwu district, Nanjing 210095, China; State Key Laboratory of Crop Genetics and Germplasm Enhancement and Utilization, Center of Agricultural Health, Academy for Advanced Interdisciplinary, Jiangsu Provincial Key Laboratory for Organic Solid Waste Utilization, Jiangsu Collaborative Innovation Center for Solid Organic Waste Resource Utilization, College of Resources and Environmental Sciences, Nanjing Agricultural University, NO. 1 Weigang, Xuanwu district, Nanjing 210095, China; School of Ecological and Environmental Sciences, East China Normal University, NO. 500 Dongchuan Street, Minghang, Shanghai 200241, China; State Key Laboratory of Crop Genetics and Germplasm Enhancement and Utilization, Center of Agricultural Health, Academy for Advanced Interdisciplinary, Jiangsu Provincial Key Laboratory for Organic Solid Waste Utilization, Jiangsu Collaborative Innovation Center for Solid Organic Waste Resource Utilization, College of Resources and Environmental Sciences, Nanjing Agricultural University, NO. 1 Weigang, Xuanwu district, Nanjing 210095, China; State Key Laboratory of Crop Genetics and Germplasm Enhancement and Utilization, Center of Agricultural Health, Academy for Advanced Interdisciplinary, Jiangsu Provincial Key Laboratory for Organic Solid Waste Utilization, Jiangsu Collaborative Innovation Center for Solid Organic Waste Resource Utilization, College of Resources and Environmental Sciences, Nanjing Agricultural University, NO. 1 Weigang, Xuanwu district, Nanjing 210095, China; School of Ecological and Environmental Sciences, East China Normal University, NO. 500 Dongchuan Street, Minghang, Shanghai 200241, China; State Key Laboratory of Crop Genetics and Germplasm Enhancement and Utilization, Center of Agricultural Health, Academy for Advanced Interdisciplinary, Jiangsu Provincial Key Laboratory for Organic Solid Waste Utilization, Jiangsu Collaborative Innovation Center for Solid Organic Waste Resource Utilization, College of Resources and Environmental Sciences, Nanjing Agricultural University, NO. 1 Weigang, Xuanwu district, Nanjing 210095, China

**Keywords:** arsenic, arsenic methylation, dimethylarsenate, straighthead disease, paddy soil, redox potential, rice

## Abstract

Microbial arsenic (As) methylation in paddy soil produces mainly dimethylarsenate (DMA), which can cause physiological straighthead disease in rice. The disease is often highly patchy in the field, but the reasons remain unknown. We investigated within-field spatial variations in straighthead disease severity, As species in rice husks and in soil porewater, microbial composition and abundance of *arsM* gene encoding arsenite *S*-adenosylmethionine methyltransferase in two paddy fields. The spatial pattern of disease severity matched those of soil redox potential, *arsM* gene abundance, porewater DMA concentration, and husk DMA concentration in both fields. Structural equation modelling identified soil redox potential as the key factor affecting *arsM* gene abundance, consequently impacting porewater DMA and husk DMA concentrations. Core amplicon variants that correlated positively with husk DMA concentration belonged mainly to the phyla of *Chloroflexi*, *Bacillota*, *Acidobacteriota*, *Actinobacteriota*, and *Myxococcota*. Meta-omics analyses of soil samples from the disease and non-disease patches identified 5129 *arsM* gene sequences, with 71% being transcribed. The *arsM-*carrying hosts were diverse and dominated by anaerobic bacteria. Between 96 and 115 *arsM* sequences were significantly more expressed in the soil samples from the disease than from the non-disease patch, which were distributed across 18 phyla, especially *Acidobacteriota*, *Bacteroidota*, *Verrucomicrobiota*, *Chloroflexota*, *Pseudomonadota*, and *Actinomycetota*. This study demonstrates that even a small variation in soil redox potential within the anoxic range can cause a large variation in the abundance of As-methylating microorganisms, thus resulting in within-field variation in rice straighthead disease. Raising soil redox potential could be an effective way to prevent straighthead disease.

## Graphical Abstract

**Graphical Abstract:**
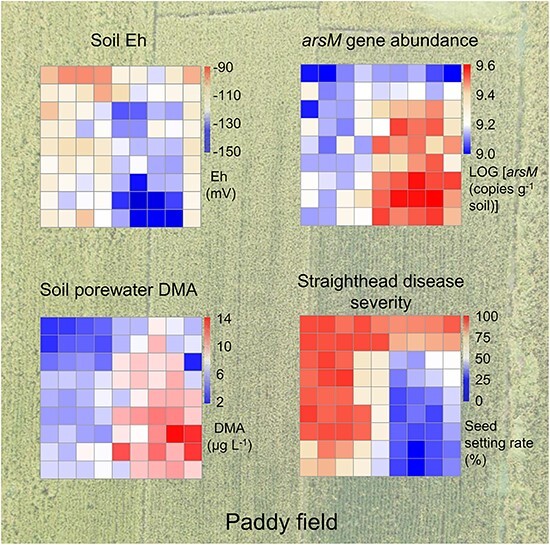

## Introduction

Soil is inherently highly heterogenous across various scales, ranging from microsites within soil aggregates to the field and regional scale [1–3]. Variation in soil properties shapes the microbial community, which in turn impacts biogeochemical processes [4]. Among the key soil properties impacting microbial communities, pH stands out prominently [5–8]. Soil redox potential, a measure of electron availability, is another key factor shaping microbial community [9–11]. Comparisons between soils maintained under oxic or anoxic conditions show vastly different microbial communities [12, 13]. Many microbial-driven biogeochemical processes are redox sensitive and are performed by specialized microbial groups within certain redox ranges, e.g. nitrification, denitrification, reduction of ferric iron, sulphate reduction, and methanogenesis [14, 15]. Some apparently non-redox sensitive transformations may also show a preference for certain redox ranges. For example, methylation of mercury is mediated primarily by anaerobes under anoxic conditions [16]. Arsenic is a redox sensitive metalloid, with arsenate [As(V)] and arsenite [As(III)] predominating under the oxic and suboxic/anoxic conditions, respectively [17]. Reduction of As(V) to As(III) renders As more mobile in the environment and more toxic to organisms. One reason why paddy rice accumulates more As than upland crops is the mobilization of As(III) in submerged paddy soil [18–21]. Arsenic is also prone to microbial methylation; a variety of methylated As and methylated thioarsenates have been detected in paddy soils and rice plants [22–26]. Flooding of soil has been found to promote As methylation [27–29], indicating that anoxic conditions favour microbial As methylation. Regional variations in the concentration and percentage of methylated As in rice have been reported [30–33], with the percentage of methylated As in total As generally increasing with latitude [26]. The reasons for this regional variation are unknown but are possibly linked to different microbial composition [33].

Many microorganisms are able to methylate As(III) to produce monomethylarsenate (MMA), dimethylarsenate (DMA), or trimethylarsine (TMA) [34]. DMA is usually the major methylated As species in both paddy soil and rice grain [28, 33]. Some microorganisms capable of As methylation have been isolated from paddy soil, including *Streptomyces* sp. [35], *Arsenicibacter rosenii* strain SM-1 [36], *Clostridium* sp. BXM [37], and *Paraclostridium* sp. EML [38]. Experiments using specific metabolic inhibitors and enrichment cultures suggested that sulphate reducing bacteria (SRB) are a major microbial group mediating As methylation in flooded paddy soil [29]. Arsenic methylation is catalyzed by the enzyme arsenite *S*-adenosylmethionine methyltransferase (ArsM) [39, 40]. ArsM proteins have a conserved SAM binding domain and two to four conserved cysteine residues, which are essential for the enzymatic activity [41, 42]. Genes (*arsM*) encoding putative ArsM proteins are widely distributed among diverse soil microorganisms [43, 44]. Microbial *arsM* genes appear to have evolved before the Great Oxidation Event on the Earth [45]. Microbial As methylation is generally considered to be a detoxification mechanism, as pentavalent MMA and DMA are less toxic to microorganisms than As(III) or As(V) and TMA(III) is volatile [17]. However, DMA appears to be much more toxic to plants than inorganic As (iAs) [46]. Both synthetic MMA and DMA were widely used as herbicides and defoliants in the past [47]. Higher plants appear to be incapable of As methylation [48], but can take up methylated As species from the soil [49]. In rice, excessive accumulation of DMA can cause straighthead disease, a physiological disorder affecting seed setting and grain yield [46, 50, 51]. This disease is prevalent in some rice growing areas, particularly in newly converted paddies and the upland-paddy rotation system [23, 52]. Straighthead disease is notably patchy within fields, and the reasons for within-field spatial variation remain unknown.

In the present study, we hypothesized that within-field variation in rice straighthead disease is related to variation in microbial As methylation in the soil. We quantified spatial variations in soil and porewater properties, porewater As species, microbial composition, *arsM* gene abundance, as well as rice straighthead disease severity and husk As species. We also used metagenomics and metatranscriptomics to help identify microorganisms associated with enhanced As methylation in the straighthead disease patches. The results reveal soil redox status as the key driver for within-field spatial variation in microbial As methylation and consequently rice straighthead disease.

## Materials and methods

### Experiments on within-field variation

Two field experiments were conducted in the rice growing season of 2021 at Tancheng (TC, Shandong province, China) and Siyang (SY, Jiangsu province, China) (Supplementary Fig. S1A), where rice straighthead disease had been observed in previous seasons. We selected an area of ~900 m^2^ of paddy field at each site, where rice straighthead disease appeared patchy in the previous season, for investigations into the spatial relationships between the soil microbial communities, microbial functional genes involved in As methylation, soil porewater As speciation, and rice straighthead disease severity. The area under investigation was divided into 9 × 9 regular grids with a 3 m distance between grids. Soil and porewater samples were collected from the 81 grid intersections at the rice booting stage, when rice panicles initiate and is a sensitive stage for the development of straighthead disease [46]. Soil redox potential (Eh) was determined at the grid intersections at the growth stages of tillering, booting, and heading. At the maturity stage, rice panicles were collected from the 153 intersections of 17 × 9 grids with a 1.5 m distance between grids within the same area, which also included the 81 grid intersections used for soil and porewater sampling. Rice was planted and managed by local farmers according to local agricultural practices. The rice cultivars Suxiu867 (subspecies *Japonica*) and Jingliangyouhuazhan (subspecies *Indica*) were grown at TC and SY sites, respectively (Supplementary Fig. S1B).

Soil Eh was measured by using a combined Pt/Ag–AgCl electrode inserted into the soil layer ~8 cm below the soil surface. Soil porewater was collected by using Rhizon porewater samplers (Rhizosphere Research Products, The Netherlands) inserted vertically into the 0–10 cm soil layer and connected with a 10-mL syringe. Each porewater sample was divided into two aliquots. An aliquot (2 mL) was used for pH determination by using a glass electrode immediately after collection. Another aliquot was acidified to pH < 2 with concentrated HCl (final HCl concentration 1%, v: v) to prevent iron precipitation and changes in As species [28]. Soil samples were collected from the 5–8 cm depth below the soil surface. Both porewater and soil samples were transported to the laboratory on dry ice and stored at −80°C for subsequent analyses. At plant maturity, six rice panicles were collected from each grid intersection. Filled or unfilled grains were separated and counted. The seed setting rate was calculated as the percentage of filled grains among all grains. Grain samples were dried at 60°C for 2 days. Husks were separated from grain samples.

### Field slope experiments

While the experiments described above utilized the natural within-field variations, additional field plots with a gentle slope were also constructed at TC and SY sites. Before rice planting, soil was tilled to 20 cm depth and puddled. A 176 m^2^ plot (22 × 8 m) was established with a 0.26^o^ slope from the irrigation inlet to the outlet, resulting in a 10 cm difference in elevation along the length of the plot (Supplementary Fig. S1C). A ridge was built surrounding the plot and covered with plastic film. Rice was planted and managed according to local agricultural practices. Soil and porewater were sampled from 28 grid intersections within the plot as described above. Rice panicles were sampled at the maturity stage from 154 grid intersections within the plot.

### DNA extraction and quantitative PCR

Total DNA in soil samples was extracted using a Power Soil DNA Isolation Kit (QIAGEN, Germany) according to the manufacturer’s instructions. The concentration and purity of DNA were measured by using a NanoDrop 2000C spectrophotometer (Thermo Scientific, Wilmington, USA). DNA was diluted and used as template for quantification of functional genes encoding *arsM* and bacterial 16S rRNA genes by using a Real-Time PCR detection System (Bio-Rad CFX96, USA). The primers used are shown in Supplementary Table S1.

### 16S rRNA gene amplicon, metagenomic, and metatranscriptomic sequencing

The compositions of bacteria and archaea in the soil samples were analysed. Bacterial and archaeal 16S rRNA genes were amplified using the primer sets 515F/907R and Arch519F/Arch 915R, respectively, targeting the V4–V5 regions. Amplicons of bacterial and archaeal 16S rRNA gene fragments were purified and quantified. Paired-end sequencing was performed on an MiSeq platform (Illumina, Shanghai Biozeron Biothchnology, Shanghai, China.). Raw sequences were quality filtered and assembled. Indel mutations and substitutions in the merged sequences were identified by the DADA2 algorithm in Quantitative Insights Into Microbial Ecology (QIIME, V1.9.0 http://qiime.org/scripts/assign_ taxonomy.html). The paired reads were trimmed and filtered with a maximum of two expected errors per read (maxEE = 2). The phylogenetic affiliation of each 16S rRNA gene sequence was identified by the RDP Classifier (http://rdp.cme.msu.edu/) against the Silva 138) database using a confidence threshold of 70%. Core ASVs (amplicon sequence variants) were defined as those present in more than 80% of the samples. Core ASVs that were significantly related to soil Eh and husk DMA concentration were identified by linear regression model with R version 4.2.1. Co-abundant groups (CAGs) of ASVs, defined as closely related and classified as a guild, were identified by co-abundant network analysis. Co-abundant network analysis was used to cluster the core ASVs which responded to the changes in soil Eh. The correlations between the core ASVs were calculated using the SparCC algorithm (bootstrap value, 100) [53]. In total, 90 and 78 core ASVs were clustered into 12 and 10 CAGs in TC and SY, respectively, by using the ward algorithm and PERMANOVA (999 permutations; *P* < 0.001) based on the SparCC correlation coefficient matrix with R version 3.1.3. The 22 CAGs with an *r* coefficient higher than 0.3 were visualized into a network with cytoscape v3.1.1 (http://www.cytoscape.org/). The abundance of each CAG was the sum of relative abundances of the ASVs within the CAG. Random forest analysis was performed to disentangle the main environmental variables and microbial contributors of husk DMA with the “randomForest” package in R (version 3.1.3) [54]. To estimate the importance of environmental variables and microbial contributors, the percentage increases in the mean squared error (MSE) of variables were calculated; higher MSE% values imply that more important variables were used [55]. The significance of each variable was assessed with the “rfPermute” package in R (version 3.1.3). Partial Least Square-Structural Equation Modelling was constructed to estimate the direct, indirect and interactive effects of the soil and porewater variables on DMA accumulation in rice husk using the R package “plspm”.

A total of 12 soil samples were selected for metagenomic and metatranscriptomic analyses, including three samples each from the straighthead disease patch and the non-disease patch at each site (Supplementary Fig. S2). Soil DNA and total RNA were extracted using Power Soil DNA Isolation Kit and Power Soil RNA Isolation Kit (QIAGEN, Germany), respectively. The extracted DNA and RNA were quantified by a NanoDrop 2000C spectrophotometer (Thermo Scientific, Wilmington, USA). RNA quality was determined using 2100 Bioanalyser (Agilent). Metagenomic shotgun sequencing libraries were constructed and sequenced at Shanghai Biozeron Biological Technology. Specifically, 1 μg genomic DNA of each sample was sheared by Covaris S220 Focused-ultrasonicator (Woburn, MA USA) and sequencing libraries were prepared with a fragment length of ~450 bp. All samples were sequenced on a HiSeq X platform (Illumina, Shanghai Biozeron Biothchnology, Shanghai, China.). High-quality RNA samples (OD260/280 = 1.8 ~ 2.2, OD260/230 ≥ 2.0, RIN ≥ 6.5, 28S/18S ≥ 1.0, total RNA > 10 μg) were used for constructing sequencing library. rRNAs were removed by Ribo-ZeroTM rRNA Removal Kits from Illumina (San Diego, CA). cDNA synthesis, end repair, A-base addition and ligation of the Illumina-indexed adaptors were performed according to the Illumina’s protocol. Metatranscriptomic sequencing was performed on an Novaseq 6000 platform (Illumina, Shanghai Biozeron Biothchnology, Shanghai, China.). On average, 30 gigabase paired-end reads were obtained for each sample.

### Trimming, functional annotation of soil metagenomes and metatranscriptomes, and population genome binning of soil metagenomes

The raw paired-end reads of metagenomic and metatranscriptomics data sets were trimmed using Multitrim pipelines (https://github.com/KGerhardt/multitrim) with default settings. SortmeRNA was used with default settings to remove residual rRNA sequences after rRNA subtraction from metatranscriptomes [56]. The high-quality clean reads were assembled into contigs using MEGAHIT (https://github.com/voutcn/megahit, version 1.1.2) with the parameters “—min-contig-len 500” [57]. Metagenome-assembled genomes (MAGs) were recovered using the contigs longer than 500 bp by MetaWRAP v1.2.1 [58]. The resulting MAGs were improved by bin_refinement model of MetaWRAP. The completeness and contamination of the refined MAGs were assessed using CheckM v1.1.3 [59] and only those MAGs with completeness ≥ 50% and contamination ≤ 10% were included in subsequent analyses. The MAGs were dereplicated using dRep v3.3.0 [60] with the parameters “-sa 0.95 -nc 0.30”. Open reading frames were predicted from contigs of each sample or MAGs by Prodigal v2.6.3 [61] with the parameters “-p meta”. Taxonomic classification of MAGs was determined by classify_wf model of GTDB-Tk v2.1.0 [62, 63]. Phylogenetic analysis of MAGs was conducted with the infer module of GTDB-Tk, and the phylogenetic tree was visualized in tvBOT v2.5.0 [64]. The abundance of the MAG was calculated by the statistic CPM (coverage per million reads). First, clean reads from each sample were mapped to the contigs of the MAG using BLAT v2.3.4.1 [65] with e-value ≤ 10^−5^, alignment of nucleotide identify of ≥ 95%, and alignment length ≥ 100 bp, and only the top hits were retained. Next, a script (http://enve-omics.ce.gatech.edu/enveomics/docs?t=BlastTab.seqdepth.pl) was used to calculate the coverage of each contig in the MAG, and the average coverage of all contigs in the MAG was estimated as the coverage of the MAG. Finally, the abundance of the MAG was calculated as the coverage of the MAG per millions of reads mapped on the MAG.

### Diversity, abundance, and transcriptional activity of *arsM* genes

To identify the diversity, abundance, and transcription of *arsM* gene, a database of *arsM* genes was constructed and manually curated. Briefly, representative ArsM sequences, which have been confirmed for the As methylation function, were retrieved from the Swiss-Prot databases (UniProt, downloaded in June 2022), and searched against the UniRef90 database (UniProt, downloaded in April 2023) using BLASTp [66] to collect the homologs of ArsM. The collected sequences were furthered manually inspected for the presence of conserved domains based on the Pfam models to determine if the sequence was truly an ArsM. Sequences lacking one or more of the conserved domains were removed from the constructed database. The predicted open reading frames from contigs or MAGs were searched against the in-house ArsM database using BLASTp [66]. Alignment of amino acid identify of ≥ 60%, query sequence length coverage of ≥ 70%, and e-value ≤ 10^−5^ was retained. MicrobeGensus was used to assess the average genome size of each sample community and calculated the total coverage of microbial genomes present in a sample, i.e. genome equivalents = total bp sequenced/average genome size in bp. The relative abundance of *arsM* genes in metagenomes was calculated by RPKG [(reads mapped to gene)/(gene length in kb)/(genome equivalents)]. The relative abundance of *arsM* genes in metatranscriptomes was calculated by the statistic reads per kb per millions of reads (RPKM, the number of reads mapping on reference sequences /gene length in kb / the total number of reads after removal of rRNA reads). The dissimilatory sulphite reductase (Dsr), anaerobic sulphite reductase (Asr), and McrA of these MAGs were also detected using BLASTp against the SCycDB database [67] and McycDB database [68], respectively, with alignment of amino acid sequences ≥ 60%, query length coverage ≥ 70%, and an e-value ≤ 10^−5^.

### Chemical analyses

Soil organic carbon (SOC) and porewater dissolved organic carbon (DOC) were determined by using an elemental analyser (TOC/TNb analyser HT1300 Analytik Jena, Germany). Soil pH (in 1: 2.5 soil: water) and porewater pH were determined using a pH electrode. Ground soil samples (< 0.15 mm) were digested with aqua regia (HCl: HNO_3_ = 4: 1) and As concentrations were determined by inductively coupled plasma mass spectrometry (ICP-MS, NexION 300X, PerkinElmer) [69]. Arsenic species in finely ground rice husk samples were extracted with 1% HNO_3_ using a microwave digester [30]. Arsenic species in soil porewater and husk extracts were quantified using high performance liquid chromatography with an anion exchange column (PRP-X100, 150 mm × 4.6 mm, Hamilton) coupled to ICP-MS operating in the helium gas collision mode.

## Results

### Within-field spatial variations in rice straighthead disease and husk DMA accumulation

**Figure 1:**
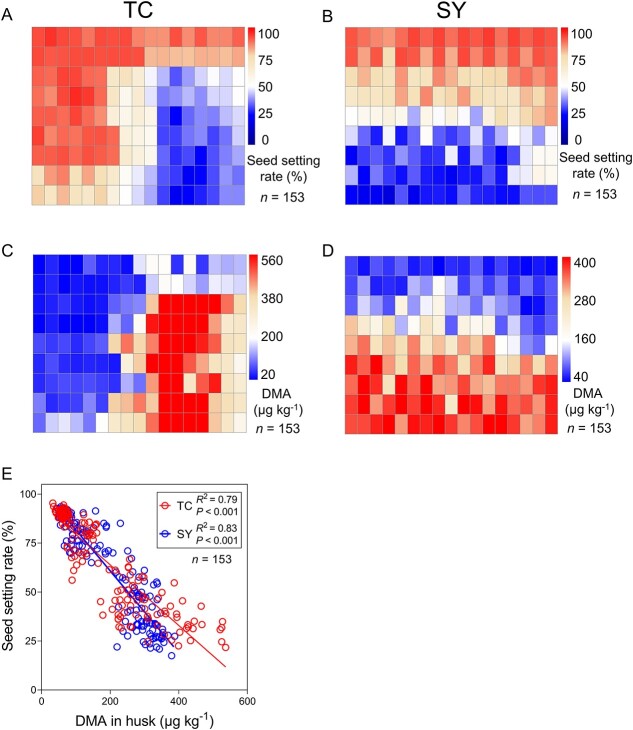
Spatial variations of the seed setting rate of rice (indicating the severity of straighthead disease) and DMA concentration in rice husk in TC and SY paddy fields. Seed setting rate in TC (A) and SY (B), and husk DMA concentration in TC (C) and SY (D) fields. The relationship between seed setting rate and husk DMA concentration (E).

We observed a large spatial variation in the degree of straighthead disease in rice plants in both TC and SY fields. Among the 153 rice panicle samples collected from regular grids at each site at the maturity stage, the seed setting rate, an indicator of straighthead disease, varied from 21.7 to 95.4% and 17.5 to 93.6% in TC and SY fields, respectively. The spatial variation in the seed setting rate is depicted as heatmaps in Fig. 1A and B. Because distortion of rice husk is a typical symptom of straighthead disease, we determined As species in the rice husk samples and found clear spatial patterns in the DMA concentration (Fig. 1C and D), which were opposite to the patterns of the seed setting rate. At both sites, there was a highly significant negative relationship between the seed setting rate and husk DMA concentration (Fig. 1E). The range of husk DMA concentration was 33.0–536.5 μg kg^−1^ in TC and 46.0–388.0 μg kg^−1^ in SY, representing 16- and 8-fold variation, respectively. Rice husks also contained 382.1–831.4 μg kg^−1^ and 645.6–939.9 μg kg^−1^ inorganic As (iAs), and 11.2–35.8 μg kg^−1^ and 18.5–45.7 μg kg^−1^ MMA in TC and SY fields, respectively. However, there were no clear patterns of spatial variation in the concentration of either iAs or MMA in the husks (Supplementary Fig. S3A-D). There was also no significant correlation between the seed setting rate and husk iAs or MMA concentration (Supplementary Fig. S3E and F).

### Within-field spatial variations in soil Eh and porewater As species

**Figure 2:**
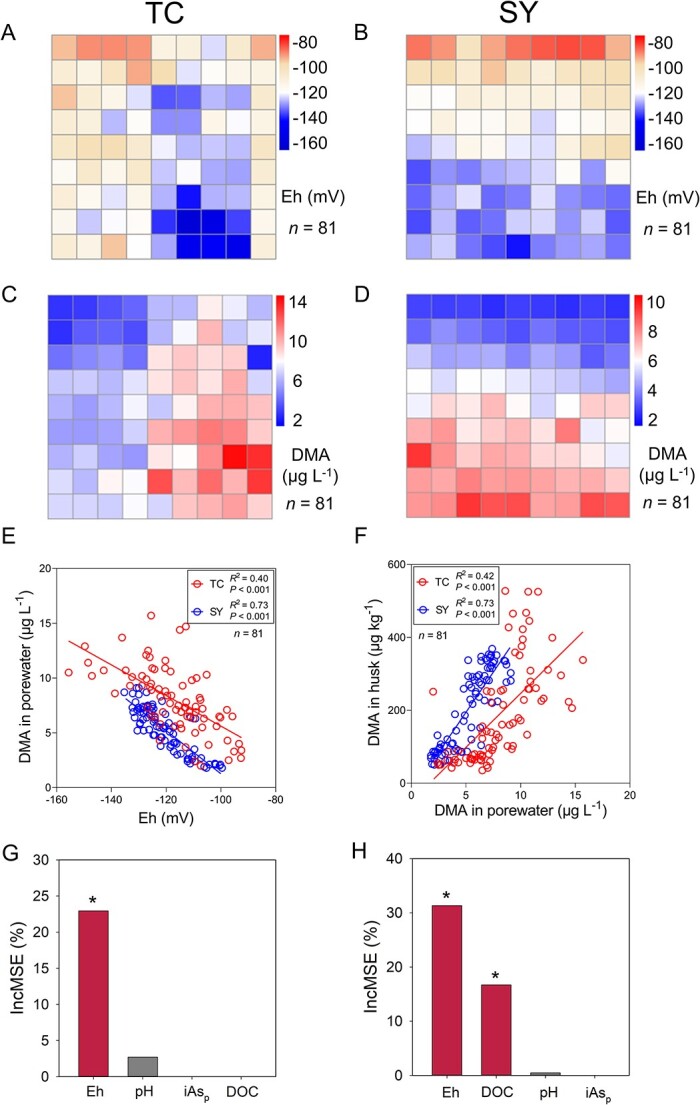
Soil redox potential as a key factor affecting porewater and husk DMA concentrations. Spatial variations in soil Eh (A, B) and porewater DMA concentration (C, D) at the rice booting stage in TC (A, C) and SY (B, D) fields. Relationship between porewater DMA concentration and soil redox potential (E), and between husk DMA concentration and porewater DMA concentration (F). The importance of factors affecting porewater DMA concentration in TC (G) and SY (H) fields based on RandomForest modelling.

At the rice growth stages of tillering, booting, and heading, we determined soil Eh at 81 grid intersections in each paddy field and also collected soil and porewater samples from the same intersections at the booting stage. There were no clear spatial patterns in either soil organic matter content, soil pH, or soil total As concentration across either field (Supplementary Fig. S4A-F), suggesting relatively even distribution of these soil properties. Soil total As concentrations (7.6–7.9 and 9.1–9.3 mg kg^−1^ in TC and SY, respectively) are within the normal background range of uncontaminated soils. We determined soil particle size distribution in 16 samples each from the straighthead disease and non-disease areas in each field and found no significant differences between the two areas (Supplementary Fig. S4G). At the tillering stage, soil Eh was below −120 mV across both fields with no clear spatial pattern (Supplementary Fig. S5), likely because the whole field was flooded with paddy water up to this growth stage. Paddy water was drained for one week at the end of the rice tillering stage, which is a common agronomic practice to control ineffective tillering, and reflooded until the late grain filling stage. At the booting stage (2 weeks after reflooding of the paddy fields), spatial variations in soil Eh emerged, ranging from −160 to −80 mV and from −140 to −100 mV at TC and SY, respectively (Fig. 2A and B). These spatial variations were likely a consequence of the uneven topography of the paddy fields, resulting in a variation in the soil water status during the draining period and subsequently variable depth of the standing water after reflooding. At the time of Eh measurement at the booting stage, the depth of paddy water varied from 3 to 7 cm, indicating a 4 cm difference in elevation across the field. The spatial variation in soil Eh was maintained at the heading stage (Supplementary Fig. S5). Both iAs and DMA were detected in the soil porewater samples collected from the two paddy fields at the booting stage, but MMA was detected only in TC samples. Neither iAs nor MMA concentrations showed a clear spatial pattern across the field (Supplementary Fig. S6). In contrast, porewater DMA concentration showed clear spatial patterns in both fields, which were opposite to the patterns of soil Eh (Fig. 2C and D). Porewater DMA concentration varied from 2.0 to 15.7 μg L^−1^ in TC and from 1.8 to 9.1 μg L^−1^ in SY, representing 7.8- and 5.0-fold variation, respectively (Fig. 2C and D), and correlated negatively with soil Eh in both fields (Fig. 2E). Furthermore, the spatial patterns of porewater DMA were consistent with those of husk DMA concentration, showing a significant positive relationship between the two variables (Fig. 2F). We used the RandomForest model to estimate the importance of environmental variables affecting porewater DMA concentration in both paddy fields. Soil Eh was the most important and significant variable affecting porewater DMA concentration in both fields. Porewater DOC was also a significant variable in SY field. Porewater pH and iAs concentrations were not significant variables (Fig. 2G and H).

### Spatial variation in *arsM* gene abundance

**Figure 3:**
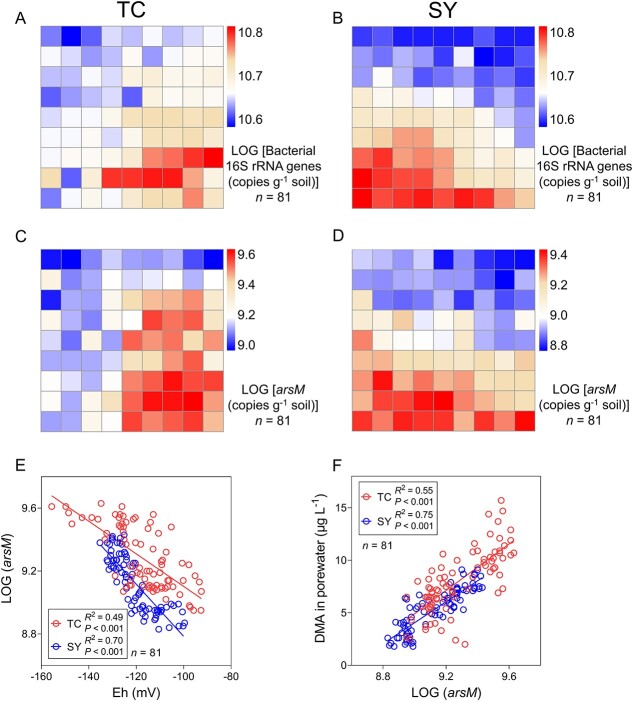
Spatial variations in the abundance of bacterial 16S rRNA and *arsM* genes. The abundance of bacterial 16S rRNA gene (A, B) and *arsM* gene (C, D) in TC (A, C) and SY (B, D) fields. Relationship between *arsM* abundance and soil redox potential (E), and between porewater DMA concentration and *arsM* abundance (F) in TC and SY paddy fields.

To investigate the cause for the variation in DMA accumulation in rice, we quantified the abundance of *arsM* and total bacterial 16S rRNA genes in the 81 soil samples collected from each paddy field at the rice booting stage using qPCR. The bacterial 16S rRNA gene abundances varied from 4.0 × 10^10^ to 6.9 × 10^10^ copies g^−1^ dry soil (1.7 fold variation) and from 4.0 × 10^10^ to 7.0 × 10^10^ copies g^−1^ dry soil (1.8 fold variation) in TC and SY fields, respectively, showing a clear spatial pattern across the field (Fig. 3A and B). The absolute abundance of *arsM* genes varied by 6.4 and 4.0 fold in TC and SY fields, respectively, and the variations were considerably larger than those of the bacterial 16S rRNA gene abundances (Fig. 3C, D). There were also clear spatial patterns in the *arsM* gene abundance across both paddy fields, with the patterns being consistent with those of porewater DMA concentration but opposite to those of soil Eh (Fig. 3C and D). The abundance of *arsM* genes showed a significant (*P* < 0.05) negative relationship with soil Eh (Fig. 3E) and a significant positive relationship with porewater DMA concentration in both paddy fields (Fig. 3F).

### Bacterial composition and relationships with husk DMA accumulation

Among the 81 soil samples, the diversity of bacterial communities based on Shannon index of bacterial 16S rRNA genes varied from 4.09 to 6.67 and from 4.28 to 6.55 in TC and SY fields, respectively (Supplementary Fig. S7A). There was a significant relationship (*R*^2^ = 0.10, *P* < 0.05) between the bacterial community diversity and soil Eh in TC, but not in SY (Supplementary Fig. S7C and D). Principal coordinate analysis based on Bray–Curtis distance of bacterial 16S rRNA gene sequences showed that the two soils are well separated by the first principal coordinate, whereas the within-field variation is manifested on the second principal coordinate (Supplementary Fig. S7B). Redundancy analysis was used to examine the relationship between the bacterial community composition and the measured soil and porewater variables. Among the explanatory variables, soil Eh had the highest correlation with the bacterial composition in both paddy fields (Supplementary Fig. S7E and F). Other variables, including DOC and porewater pH, also showed a significant correlation.

**Figure 4:**
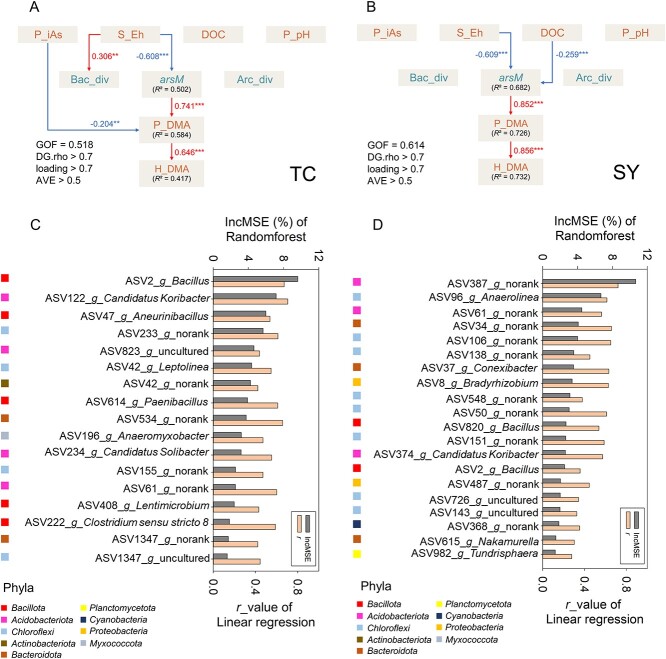
Environmental variables and amplicon sequence variants (ASVs) affecting rice husk DMA concentration. Partial Least Square-structural equation modelling showing the effects of environmental variables on husk DMA concentration in TC (A) and SY (B) fields. Identification of ASVs that were related to husk DMA concentration by linear regression and RandomForest model in TC (C) and SY (D) fields.

We further used Partial Least Square-Structural Equation Modelling to describe direct and/or indirect interaction pathways among environmental variables, *arsM* gene abundance, and bacterial diversity (Shannon index) affecting DMA accumulation in rice husk. A goodness of fit of 0.518 and 0.614 was obtained for the TC and SY models, respectively, which explained 41.7% and 73.2% of the variance in husk DMA concentration in TC and SY, respectively (Fig. 4A and B). It is clear from both TC and SY models that the most important effect path was soil Eh directly impacting *arsM* abundance, which in turn impacted soil porewater DMA concentration, leading to variation in husk DMA concentration. Other effects, such as porewater DOC on *arsM* gene abundance, and porewater iAs on porewater DMA, were relatively small and observed only in one of the two fields. A significant effect of soil Eh on bacterial diversity was observed only in TC.

Linear regression was used to identify core ASVs that were significantly and negatively related to soil Eh. The ASVs identified were clustered into 12 and 10 co-abundant groups (CAGs) in TC and SY soils, respectively. The CAGs containing ASVs with *r* > 0.30 between each other are displayed in a co-abundant network (Supplementary Fig. S8). The abundance of these CAGs increased with decreasing soil Eh, i.e. the more negative soil Eh the higher abundance of the CAGs (Supplementary Figs S9 and S10). Furthermore, 6 and 7 core ASVs were found to correlate negatively with soil Eh (*r* > 0.60, *P* < 0.05) and positively with husk DMA concentration (*r* > 0.60, *P* < 0.05, Supplementary Fig. S11) in TC and SY, respectively. These ASVs belong to the genera of *Leptolinea*, *Bacillus*, *Candidatus Koribacter, Aneurinibacillus*, *Anaeromyxobacter*, *Anaerolinea*, *Conexibacter*, *Bradyrhizobium* and uncultured genera (Fig. 4 and Supplementary Fig. S11). Furthermore, we used the Randomforest model to identify important ASVs that were related to husk DMA concentration (*R*^2^ = 0.51 and 0.58 for TC and SY, respectively). Sixty-four and 56 ASVs were identified with a significant (*P* < 0.05) contribution to the variation in husk DMA accumulation for TC and SY fields, respectively, which belong to the phyla of *Acidobacteriota*, *Actinobacteriota*, *Bacteroidota*, *Chloroflexi*, *Firmicutes*, *Proteobacteria*, *Planctomycetota*, *Myxococcota*, *Desulfobacterota*, and others (Supplementary Fig. S12). Among them, 18 and 20 ASVs were also identified with significantly negative correlation with soil Eh by linear regression in TC and SY, respectively (Fig. 4C and D). These ASVs were classified into the genera of *Leptolinea*, *Candidatus Koribacter*, *Bacillus*, *Aneurinibacillus*, *Anaeromyxobacter*, *Paenibacillus*, *Lentimicrobium*, *Clostridium sensu stricto 8*, *Candidatus Solibacter*, *Bradyrhizobium*, *Conexibacter*, *Anaerolinea*, *Nakamurella*, *Tundrisphaera*, norank and uncultured (Fig. 4C, D).

### Microbial composition and expression of *arsM* genes in normal and straighthead disease patches

To examine the diversity and transcriptional level of *arsM* genes, we selected three soil samples each from the patches with severe straighthead disease and without disease in each field for metagenomic and metatranscriptomic analyses (Supplementary Fig. S2). A total of 5129 *arsM* gene sequences were identified from metagenomic analysis in the TC and SY samples. Taxonomic classification of the identified *arsM* genes showed that they were diverse and distributed in bacteria (92%) and archaea (8%). *Actinomycetota*, *Pseudomonadota*, *Bacteroidota*, *Acidobacteriora*, and *Chloroflexota* were the five most frequently detected phyla harbouring *arsM* genes (Supplementary Fig. S13). Compared with the soil samples from the non-disease patch, those from the disease patch contained a significantly higher abundance (*P* < 0.05) of eight and six *arsM* gene sequences in TC and SY, respectively. The eight hosts harbouring *arsM* gene in TC were classified into *Microbacterium*, *Geodematophilus*, *Krasilnikovia* and two unclassified genera of *Actinomycetes* class, *Longilinea* genus of *Anaerolineales* class, *Thermoleophilia* class, and *Acidobacteriota* phylum (Supplementary Fig. S14A). The six hosts harbouring *arsM* gene in SY are classified into *Candidatus Koribacter* and unclassified class of *Acidobacteriota* phylum, *Thermoleophilia* class, *Nocardiodaceae* family of *Actinomycetota* phylum, *Hyphomicrobiales* order, and *Gemmatimonadota* phylum (Supplementary Fig. S14B). Two hosts harbouring *arsM* gene belonging to *Actinomycetota* and *Acidobacteriota* phyla are shared between TC and SY soils (Supplementary Fig. S14C and D).

**Figure 5:**
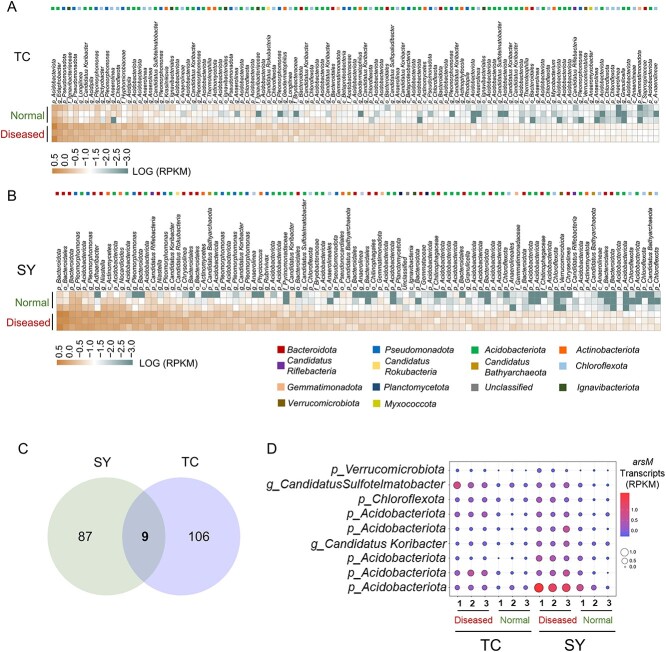
Metatranscriptomics reveal the differentially expressed *arsM* genes between the straighthead disease and non-disease patches. The abundance of *arsM* gene transcripts in the disease and non-disease patches in TC (A) and SY (B) fields. Only those with a log(RPKM) > 10^−2^ in the disease patch are shown. Venn diagram showing the number of differently expressed *arsM* genes (C) and their host species (D) shared between TC and SY fields.

Metatranscriptomic analysis showed that 71% of the identified *arsM* genes in the metagenomic datasets were transcribed, among which 96 and 115 (RPKM > 0.01) were significantly (*P* < 0.05) more expressed in the soil samples from the disease patch than those from the non-disease patch in TC and SY, respectively (Fig. 5A and B). The differentially expressed *arsM* genes were distributed across 18 phyla including *Acidobacteriota*, *Bacteroidota*, *Verrucomicrobiota*, *Chloroflexota*, *Pseudomonadota*, *Actinomycetota*, *Bacillota*, *Gemmatimonadota*, *Candidatus Bathyarchaeota*, *Candidatus Cloacimonetes*, *Candidatus Riflebacteria*, *Candidatus Rokubacteria*, *Candidatus Thermoplasmatota*, *Euryarchaeota*, *Ignavibacteriota*, *Myxococcota*, *Nitrospirota*, and *Planctomycetota*, with the first five phyla accounting for 83.5–87.4% of all differentially expressed *arsM* genes (Fig. 5A and B). Nine differentially expressed *arsM* genes were shared in the two paddy soils and classified into the phyla of *Acidobacteriota*, *Chloroflexota*, and *Verrucomicrobiota* (Fig. 5C and D).

### Identification of metagenome-assembled genomes (MAGs) harbouring *arsM* genes

**Figure 6:**
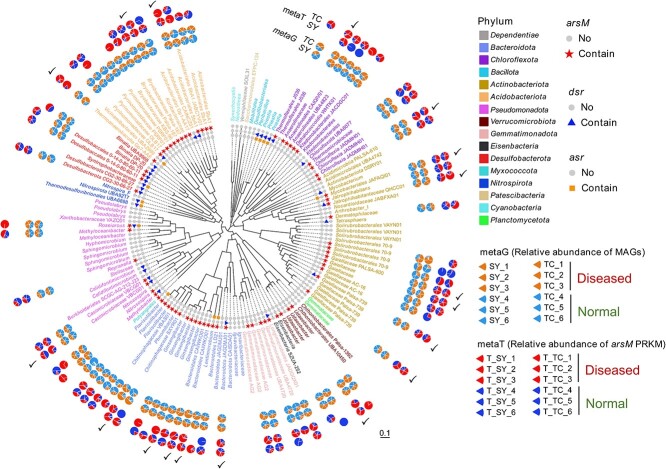
The presence and expression of *arsM* gene in metagenome-assembled genomes (MAGs). *arsM*, *dsr*, and *asr* genes were found to coexist in MAGs via blast against the database. Star, triangle, and square represent the presence of *arsM*, *dsr*, and *asr* genes in MAGs. The relative abundance of *arsM* gene and transcript in the straighthead disease and non-disease patches is displayed using pie charts. Ticks represent *arsM* genes that were more expressed in the straighthead disease patches in both TC and SY fields. The scale (0.1) indicates sequence divergence.

In total 156 metagenome-assembled genomes (MAGs) were binned from the 12 metagenomic datasets with completeness ≥ 50% and contamination ≤ 5%, among which 139 were annotated as bacteria and 17 as archaea. The bacterial MAGs belong to the phyla of *Bacteroidota*, *Chloroflexota*, *Bacillota*, *Actinobacteriota*, *Acidobacteriota*, *Pseudomonadota*, *Verrucomicrobiota*, *Gemmatimonadota*, *Eisenbacteria*, *Desulfobacterota*, *Myxococcota*, *Nitrospirota*, *Patescibacteria*, *Cyanobacteria*, *Planctomycetota*, and *Dependentiae* (Fig. 6), whereas the archaeal MAGs belong to the phyla of *Thermoproteota*, *Halobacteriota*, *Thermoplasmatota*, and *Methanobacteriota* (Supplementary Fig. S15). Of the 156 MAGs, only 23 contained genes encoding cytochrome C oxidase, a genetic marker for aerobes [70], indicating a relative low fraction of aerobes of the total MAGs; the remaining MAGs (85%) were possible anaerobes. A total of 86 distinct *arsM* genes were identified in MAGs, among which 73 and 13 were associated with bacteria and archaea, respectively (Fig. 6 and Supplementary Fig. S15). Most (93%) of the *arsM* genes were not found to coexist with the cytochrome C oxidase genes (i.e. likely anaerobes). In addition, *arsM* and *dsr* genes were found to coexist in 15 MAGs, whereas *arsM* and *asr* (encoding anaerobic sulphite reductase) genes were found to coexist in five MAGs, suggesting a relatively high frequency of *arsM* genes being present in sulphate-reducing bacteria.

We calculated the relative abundance (RPKM) of the transcribed *arsM* genes based on the metatranscriptomic datasets. Most (95.3%) of the *arsM* genes in MAGs were transcribed, including 50 *arsM* genes that were expressed in both TC and SY samples and 32 expressed in either soil. In addition, 18 *arsM* genes in MAGs were significantly (*P* < 0.05) more expressed in the soil samples from the disease patch than in those from the non-disease patch in both SY and TC. These *arsM* genes were distributed in the phyla of *Chloroflexota*, *Actinobacteriota*, *Verrucomicrobiota*, *Bacteroidota*, *Pseudomonadota*, *Acidobacteriota*, and *Halobacteriota*. Fourteen *arsM* genes in distinct MAGs were more expressed in the disease patch only in TC, which were hosted in the phyla of *Chloroflexota*, *Actinobacteriota*, *Verrucomicrobiota*, *Bacteroidota*, *Pseudomonadota*, *Desulfobacterota*, *Halobacteriota*, *Thermoproteota*, and *Methanobacteriota*. Five *arsM* genes were more expressed in the disease patch than in the non-disease patch only in SY, which were distributed in the phyla of *Gemmatinonadota*, *Desulfobacterota*, *Acidobacteriota*, and *Thermoproteota* (Fig. 6 and Supplementary Fig. S15).

### Field slope causes variations in soil Eh, DMA accumulation, and rice straighthead disease

The experiments described above represent natural variations within paddy fields. To confirm the effect of soil Eh on As methylation and rice straighthead disease, we conducted field slope experiments at TC and SY sites by establishing field plots (22 m × 8 m) with a gentle slope (0.26^o^). Irrigation and draining of paddy water generated a gradient of soil Eh values along the slope, varying from −174 to −125 mV in TC and from −138 to −116 mV in SY at the rice booting stage (Supplementary Fig. S16A and B). Analysis of 28 soil samples collected from regular grid intersections in each field slope showed reasonably homogenous distribution of soil total As and iAs in porewater (Supplementary Fig. S16C-F). In contrast, porewater DMA concentration showed a spatial pattern increasing from the top of the slope to the bottom of the slope in both fields (Supplementary Fig. S16G and H), which was opposite to the spatial pattern of soil Eh. The abundance of *arsM* genes showed a similar spatial pattern as the DMA concentration in porewater (Supplementary Fig. S16I and J). At the rice maturity stage, 154 rice samples were collected from regular grids across the slope and analysed for As species in the husk. Husk DMA concentration exhibited a clear spatial pattern increasing from the top to the bottom of the slope (Supplementary Fig. S17A and B), whereas the seed setting rate decreased in the same direction (Supplementary Fig. S17C and D). In contrast, there was no clear spatial pattern in husk iAs concentration (Supplementary Fig. S17E and F). Thus, the data from the artificial slope experiments are consistent with those of the natural within-field variation.

## Discussion

Within-field variations in soil properties and microbial community are well recognized [1, 2, 71]. In the case of paddy soil, the irrigation and management of paddy water can generate an additional layer of spatial variation by affecting the soil redox potential, which is a master variable affecting many biogeochemical processes in soil [14]. Spatial variation is often a hindrance to investigations into soil properties and function. However, within-field spatial variation also provides an opportunity to investigate key factors controlling biogeochemical processes. In the present study, we took advantage of within-field variation in rice straighthead disease to investigate key factors related to this physiological disorder. We found a highly significant relationship between the severity of straighthead disease and husk DMA concentration, but not with iAs concentration, supporting the notion that DMA accumulation causes straighthead disease in rice [46, 50, 51]. Furthermore, we found that soil redox potential was the dominant factor driving within-field variations in microbial *arsM* gene abundance and soil porewater DMA concentration, consequently impacting husk DMA concentration and the severity of straighthead disease. The soils under investigation contained only background levels of As, which was also relatively uniformly distributed across the fields, suggesting that it is microbial transformation of As species, not total As concentration, that determines the occurrence of straighthead disease.

It has been shown that anoxic conditions developed after soil is flooded promotes As methylation [72–74]. There are several possible explanations for this effect [28]. First, anoxic conditions lead to mobilization of As(III), the substrate for As methylation, due to microbial reduction of As(V) to the more mobile As(III) and also reductive dissolution of iron oxyhydroxides which releases the sorbed As species [20, 21, 75]. Second, the ability to methylate As may be more common in anaerobic microorganisms than in aerobes. In the present study, soil Eh was < −80 mV across the paddy fields, which was already lower than the threshold redox range for As(V) and Fe(III) reduction in the soil redox ladder [14]. This explains why there was no clear spatial pattern in porewater iAs concentration. Thus, the availability of As(III) was not the limiting factor for microbial methylation across the fields. The relatively small Eh variation (−40 and − 80 mV at SY and TC, respectively) generated a large variation in the abundance of *arsM* genes (4- and 6.4-fold at SY and TC, respectively), suggesting that the *arsM* gene carrying microorganisms are sensitive to small variations in the soil redox status within the anoxic range. Moreover, the variation in *arsM* gene abundance was considerably larger than the abundance in bacterial 16S rRNA genes (1.6-fold), suggesting that, as soil Eh decreases, the *arsM* gene carrying microorganisms are preferentially stimulated. Thus, increased microbial population capable of As methylation is the main reason for enhanced As methylation under anoxic conditions. Indeed, *arsM* genes were more common in the anaerobes (i.e. MAGs without cytochrome C oxidase genes, 60%) than in those of aerobes (26%) as revealed in the paddy soils herein studied. Although the absence of cytochrome C oxidase genes in MAGs could also be due to the incompleteness of the binned MAGs, it is reasonable to assume that most of these MAGs are associated with anaerobes since they are dominant in anoxic paddy soils [14]. Based on 16S rRNA gene amplicon sequencing, a number of bacterial ASVs were found to be enhanced by decreasing soil Eh and correlated significantly and positively with husk DMA concentration across the two fields. The majority of these ASVs belong to the phyla of *Choroflexi*, *Bacillota*, *Acidobacteriota*, *Bacteroidota*, *Actinobacteriota*, *Proteobacteria*, and *Myxococcota*. It is possible that these bacteria contributed to the within-field spatial variation in As methylation.

Metagenomic and metatranscriptomic analyses revealed a large number of *arsM* gene sequences with the majority also being expressed. Most of the *arsM* genes are hosted in bacteria with archaea contributing to a relatively small percentage. Among the 156 MAGs, over half (55%) contain an *arsM* gene sequence, suggesting a widespread occurrence of *arsM* genes in the anaerobes of the two paddy soils, which may explain the high As methylation potential of the two soils and the high susceptibility of rice straighthead disease. The *arsM* gene hosts are phylogenetically diverse, with up to 20 phyla containing *arsM* genes; the majority of *arsM* genes were found in the phyla of *Actinobacteriota*, *Bacteroidota*, *Acidobacteriota*, and *Chloroflexota*. Based on metatranscriptomic analysis, we also identified a large number (313) of *arsM* genes that were significantly more expressed in the straighthead disease patch than in the non-disease patch. Only a small percentage (< 4%) of these differentially expressed *arsM* genes were shared between the TC and SY fields, again indicating the diverse nature of As methylating microorganisms in paddy soils. The differentially expressed *arsM* genes were mainly associated with the phyla of *Actinobacteriota*, *Bacteroidota*, *Acidobacteriota*, and *Chloroflexota* phyla. A previous study based on metabolic inhibitors and enrichment cultures suggested that sulphate-reducing bacteria are involved in As methylation in anoxic paddy soils [29]. Among the MAGs containing *arsM* genes, about half also harbour a *dsr* or an *asr* gene, suggesting that SRB are an important group of anaerobes for As methylation in paddy soil. However, only a few differentially expressed *arsM* genes between the straighthead disease and non-disease patches are hosted by SRB, suggesting that other anaerobes may play a more important role in causing within-field spatial variation in As methylation.

Soil porewater DMA concentration is determined by both the As methylation and demethylation processes [29]. Although many microorganisms are able to methylate As, some microorganisms can demethylate methylated As compounds. For example, some methylotrophic methanogens can demethylate DMA [76]. Given that methanogens are active under highly anoxic conditions, it may be expected that decreasing redox potential within the anoxic range would also enhance As demethylation, resulting in decreased DMA concentration. The observation that porewater DMA concentration increased with decreasing soil Eh in the present study suggests that soil redox status affects As methylation more than demethylation.

In conclusion, we have identified soil redox status as the key driver controlling microbial As methylation in paddy soil. Even a relatively small decrease in soil Eh within the anoxic range can cause a large increase in microbial *arsM* gene abundance, consequently impacting DMA accumulation and the incidence of straighthead disease in rice. Previous studies regarding the impact of soil redox status on microbial community often focused on comparisons between oxic and anoxic conditions [10, 12, 13]. Our study reveals that even a relatively small change in Eh can produce a large effect on soil microbial community with profound consequences for biogeochemical processes. Our metagenomic and metatranscriptomic analyses also show that As methylating microorganisms are diverse in paddy soils. Based on our findings, raising soil Eh through agronomic practices such as periodic draining of paddy water, should offer a practical strategy to limit microbial As methylation and prevent rice straighthead disease. Indeed, some earlier field studies reported the effectiveness of mid-season draining in controlling straighthead disease, albeit without a clear understanding of the underlying mechanisms [77, 78]. This study sheds light on the mechanism behind this effect.

## Supplementary Material

Supplementary_Tables_and_Figures_wrae057

## Data Availability

The raw sequences of 16S rRNA genes have been deposited in the NCBI SRA database under the accession numbers PRJNA1065873 for bacteria and PRJNA1066387 for archaea. The metagenome and metatranscriptome data are deposited into the NCBI Genome database under the bioproject of PRJNA1065390 and PRJNA1065581, respectively.
