# Supplementary material for: Soil redox status governs within-field spatial variation in microbial arsenic methylation and rice straighthead disease
Source: ISME J. 2024 Apr 2;18(1):wrae057. doi: 10.1093/ismejo/wrae057 (PMC11031232; doi:10.1093/ismejo/wrae057)

Soil redox status governs within-field spatial variation in microbial arsenic methylation and rice straighthead disease

A-Xiang Gao[1, †], Chuan Chen[1, †], Zi-Yu Gao[2], Zhi-Qiang Zhai[1], Peng Wang[1], Si-Yu Zhang[2*] and Fang-Jie Zhao[1*]

[1] State Key Laboratory of Crop Genetics and Germplasm Enhancement and Utilization, Center of Agricultural Health, Academy for Advanced Interdisciplinary, Jiangsu Provincial Key Laboratory for Organic Solid Waste Utilization, Jiangsu Collaborative Innovation Center for Solid Organic Waste Resource Utilization, College of Resources and Environmental Sciences, Nanjing Agricultural University, Nanjing 210095, China

[2] School of Ecological and Environmental Sciences, East China Normal University, Shanghai, China

* Author for correspondence: Fang-Jie Zhao, Email: Fangjie.Zhao@njau.edu.cn

Si-Yu Zhang, Email: syzhang@des.ecnu.edu.cn

[†] These authors contributed equally.

Supplementary Tables and Figures

Table S1. The primers used in the present study.

q-PCR for *arsM*	*arsM*-309F	GYIWWNGGIVTNGAYATGA
	arsM-470R	ARRTTIAYIACRCARTTNS
q-PCR for bacterial 16S rRNA genes	BACT1369F	CGGTGAATACGTTCYCGG
	PROK1492R	GGWTACCTTGTTACGACTT
Sequencing for bacterial 16S rRNA genes	515F	GTGCCAGCMGCCGCGG
	907R	CCGTCAATTCMTTTRAGTTT
Sequencing for archaeal 16S rRNA genes	Arch519F	CAGCCGCCGCGGTAA
	Arch915R	GTGCTCCCCCGCCAATTCCT

Figure S1. Layout of field experiments in TC and SY paddy fields. Locations of the field experiments (A). Sampling points of rice (n = 153) and paddy soil and porewater (n = 81) in TC and SY fields (B). (C) A diagram showing the slope plot experiments in TC and SY fields. The slope was created from the irrigation inlet to the outlet. Red circles represent the sampling sites of soil and porewater samples, black crosses the sampling sites of rice panicles.

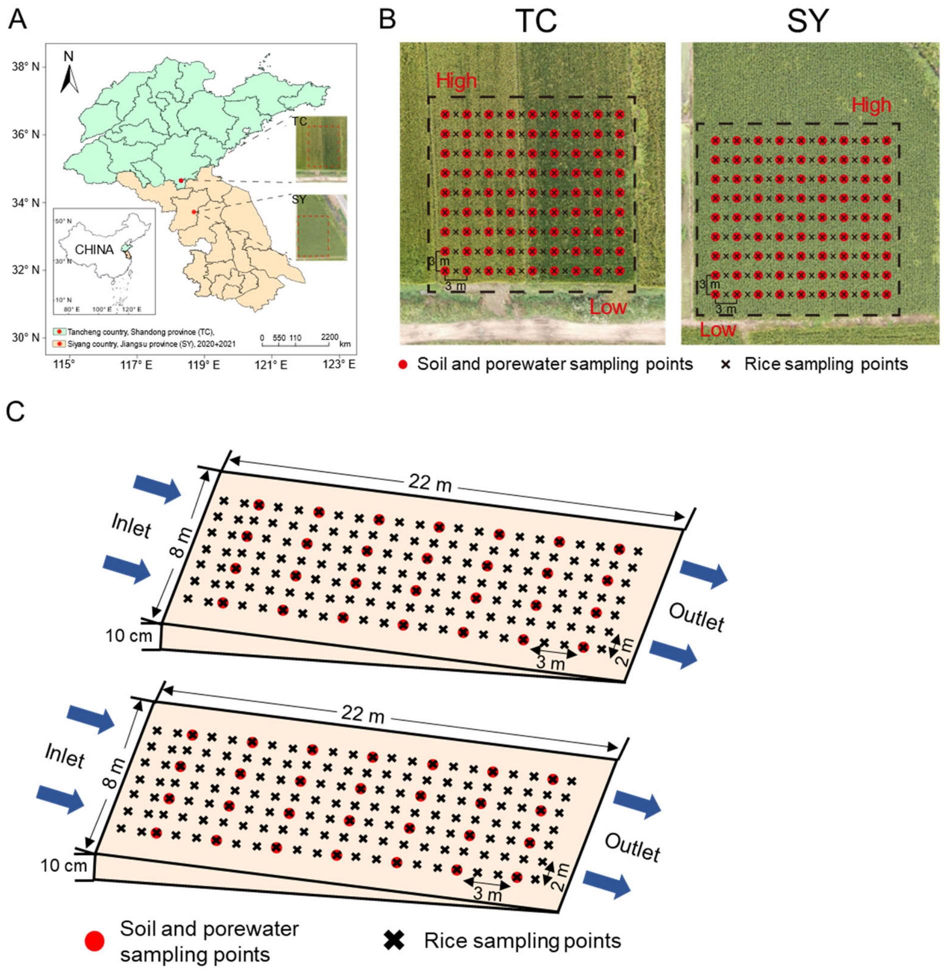

Figure S2. Soil samples from the straighthead disease and non-disease patches selected for metagenomic and metatranscriptomic analysis in TC (A) and SY (B) fields. Black and yellow circles represent the samples from the non-disease and disease patches, respectively.

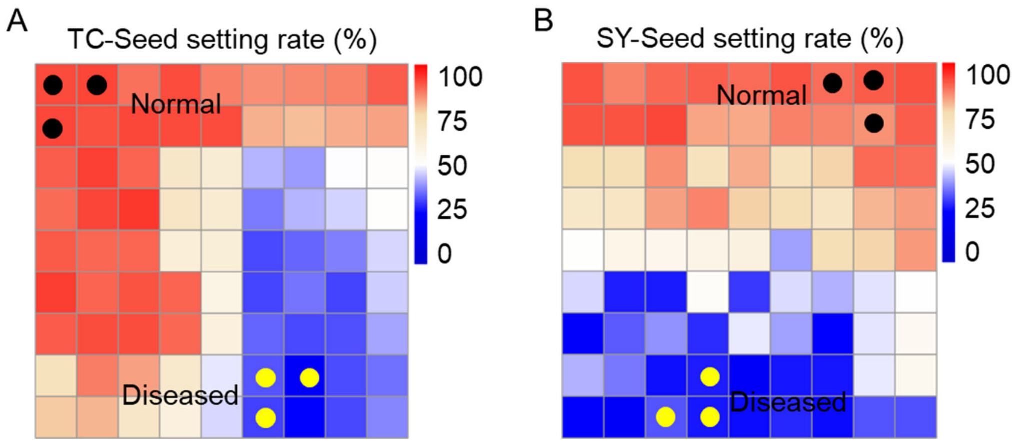

Figure S3. Spatial variations in As species in rice husk: concentrations of iAs (A, B) and MMA (C, D) in TC (A, C) and SY (B, D) fields. Relationships between seed setting rate and iAs (E) and MMA (F) concentration in rice husk.

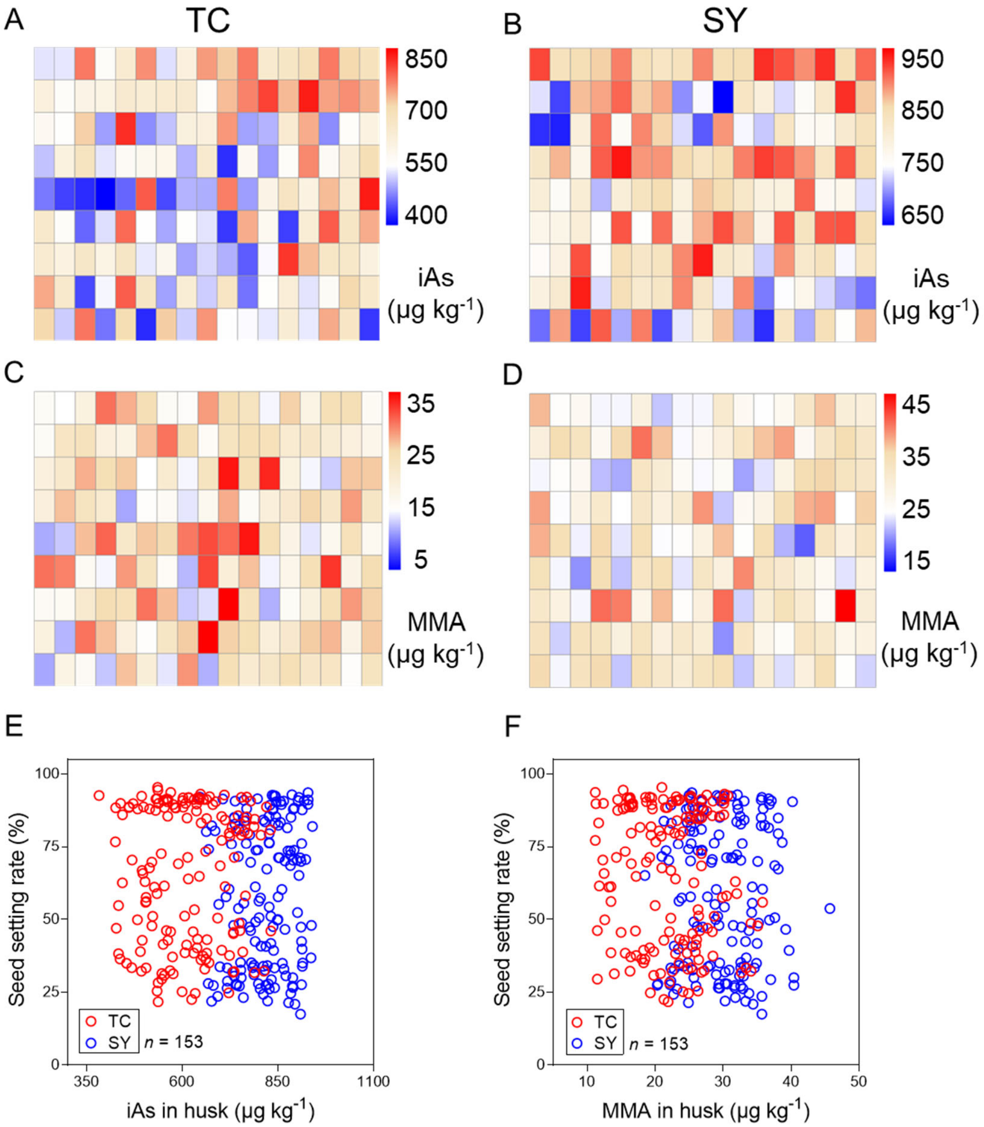

Figure S4. Spatial variations in soil total As concentration (A, B), soil pH (C, D) and soil organic matter content (E, F) in TC (A, C, E) and SY (B, D, F) fields. Particle size distribution of soils collected from non-disease and disease patches in TC and SY fields (G). ★ in (A) and (B) denotes the sampling sites of soils used for particle size analysis.

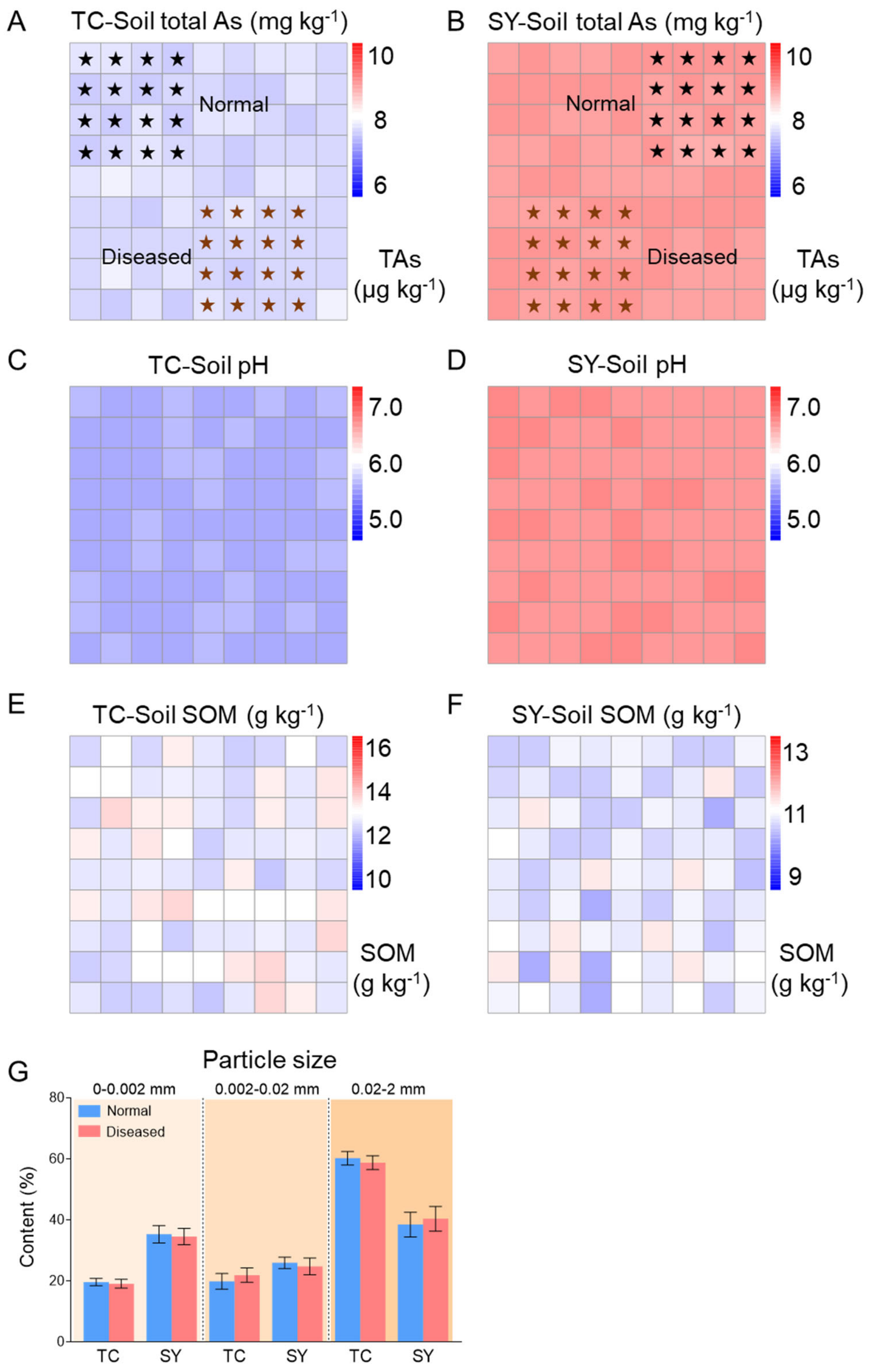

Figure S5. Spatial variations in soil Eh at the rice growth stages of late tillering and heading in TC (A) and SY (B) paddy fields.

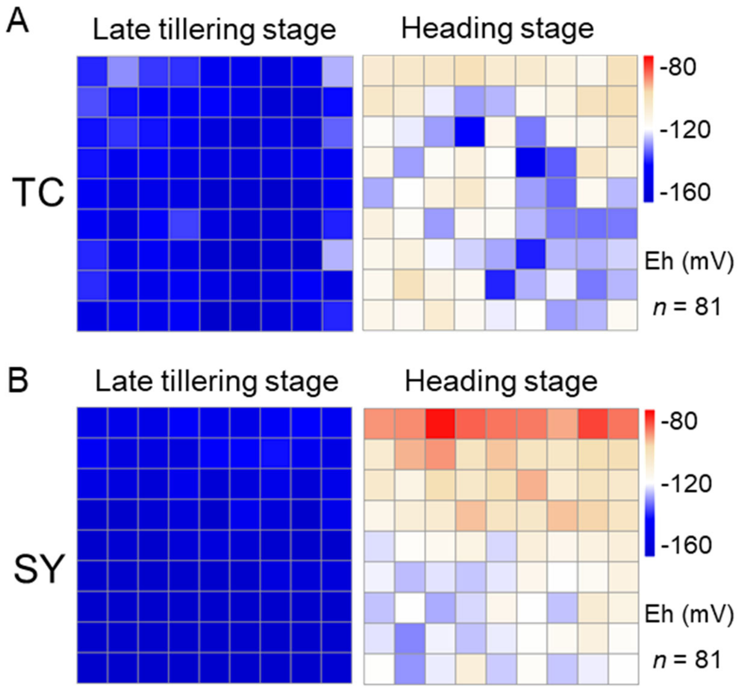

Figure S6. Spatial variations in As species in porewater. Spatial variation in iAs concentration (A, B) in porewater collected from TC (A) and SY (B) fields, and MMA concentration (C) in porewater from TC field.

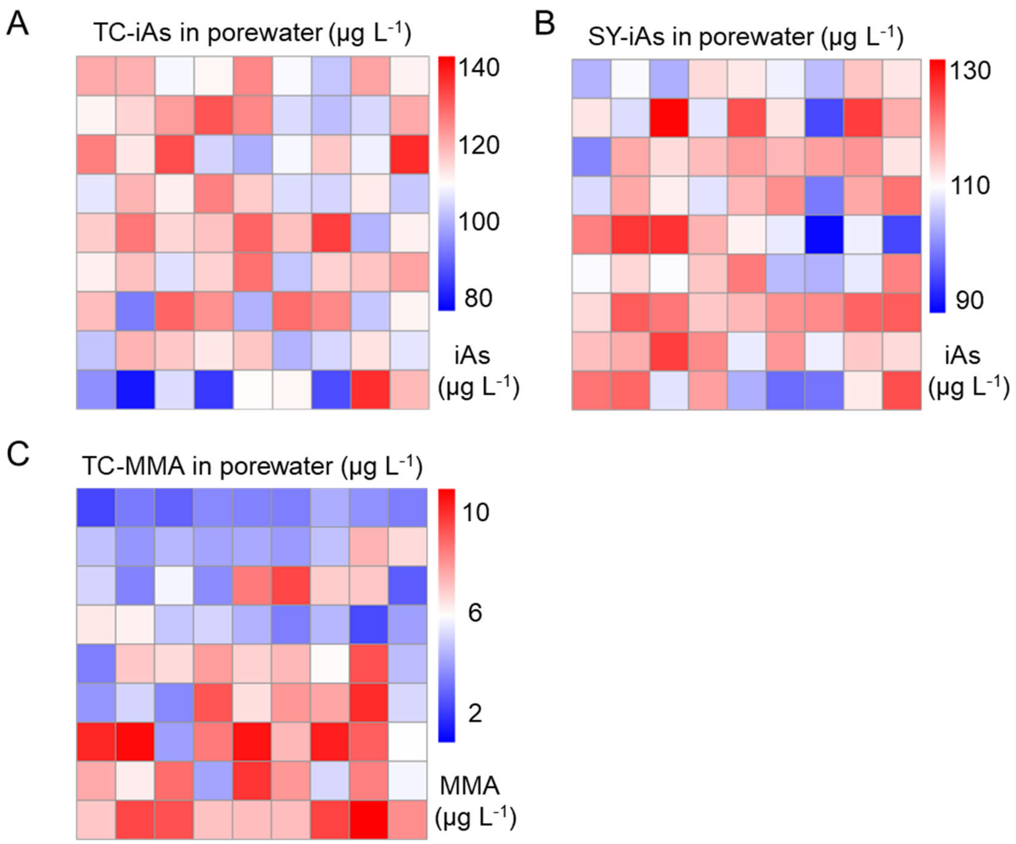

Figure S7. Diversity of bacterial compositions in TC and SY soils. Shannon index (A) and Principal coordinate analysis based on Bray-curtis distance (B) of bacterial compositions. Relationship between soil Eh at the booting stage and Shannon index of TC (C) and SY (D) soils. Redundancy analysis showing the effect of environmental variables on bacterial compositions in TC (E) and SY (F) soils.

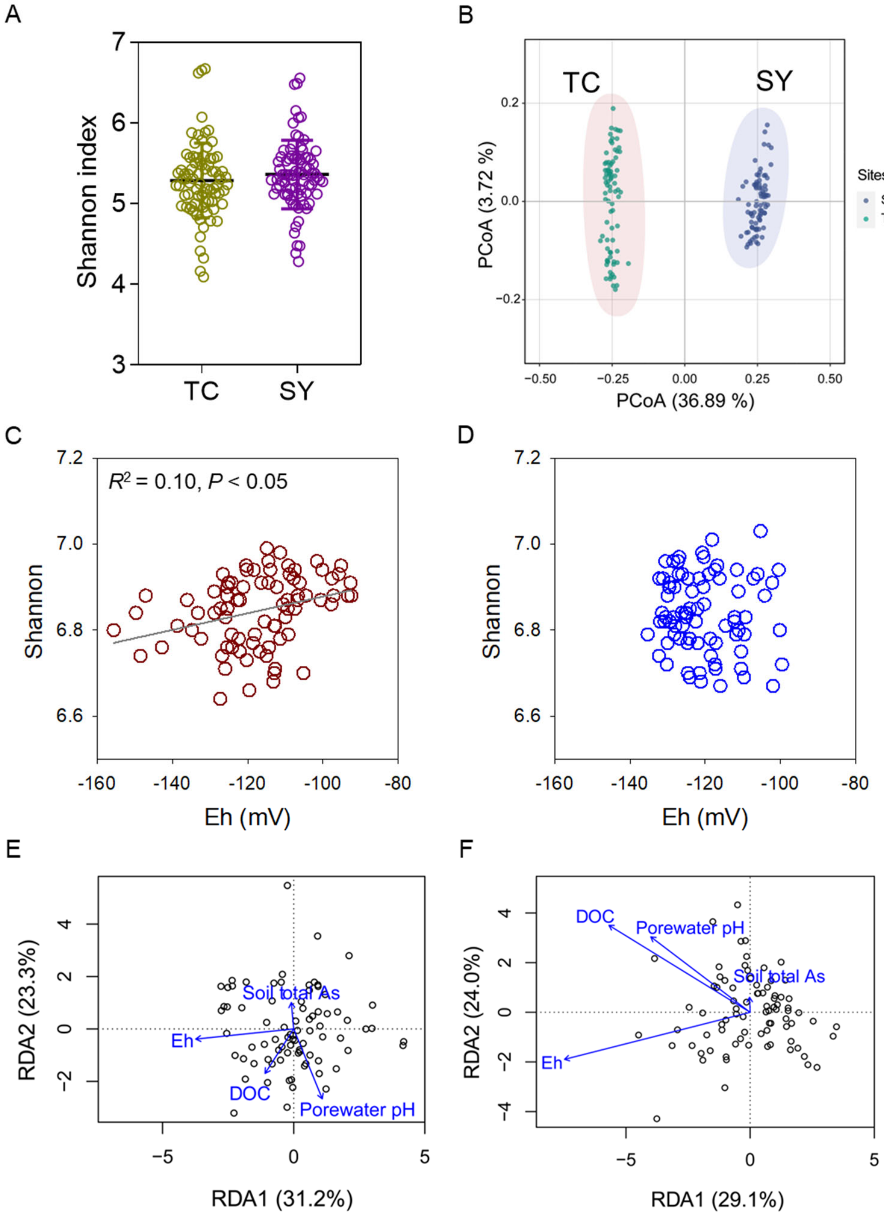

Figure S8. Co-abundant network analysis of core ASVs that were negatively related to soil Eh in TC (A) and SY (B) soils.

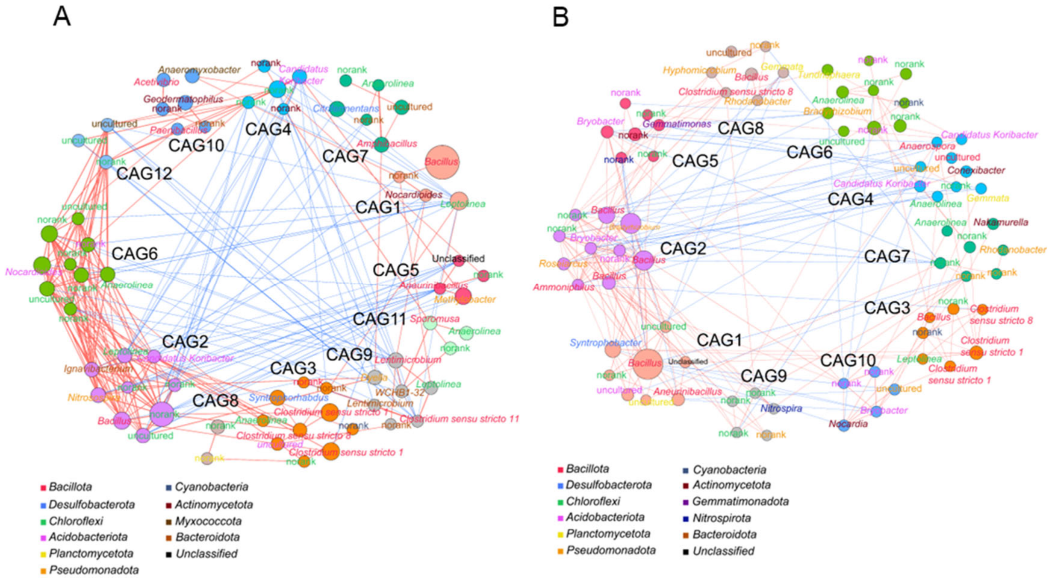

Figure S9. Relationship between co-abundant groups (CAGs) of ASVs and soil Eh in TC soil.

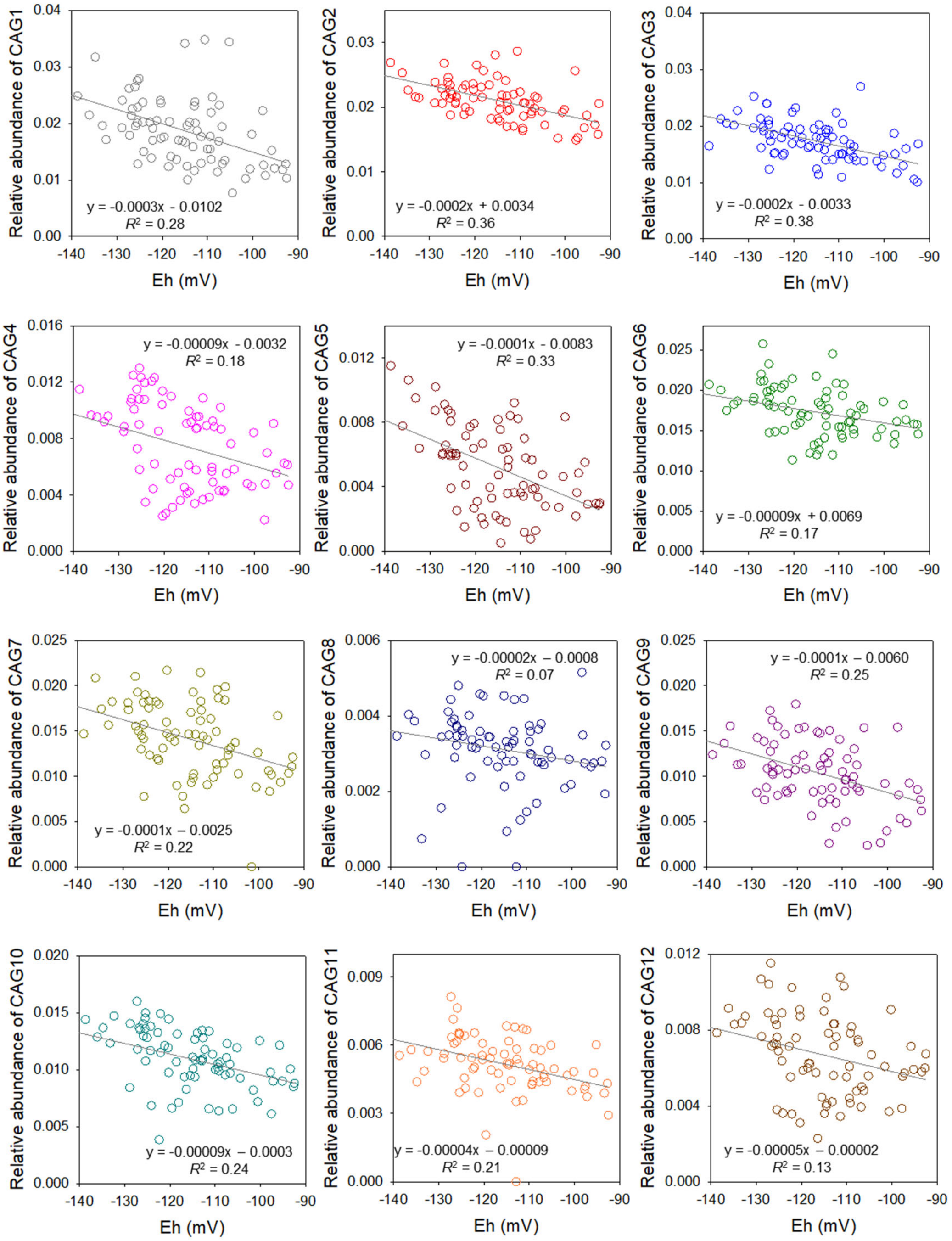

Figure S10. Relationship between co-abundant groups (CAGs) of ASVs and soil Eh in SY soil.

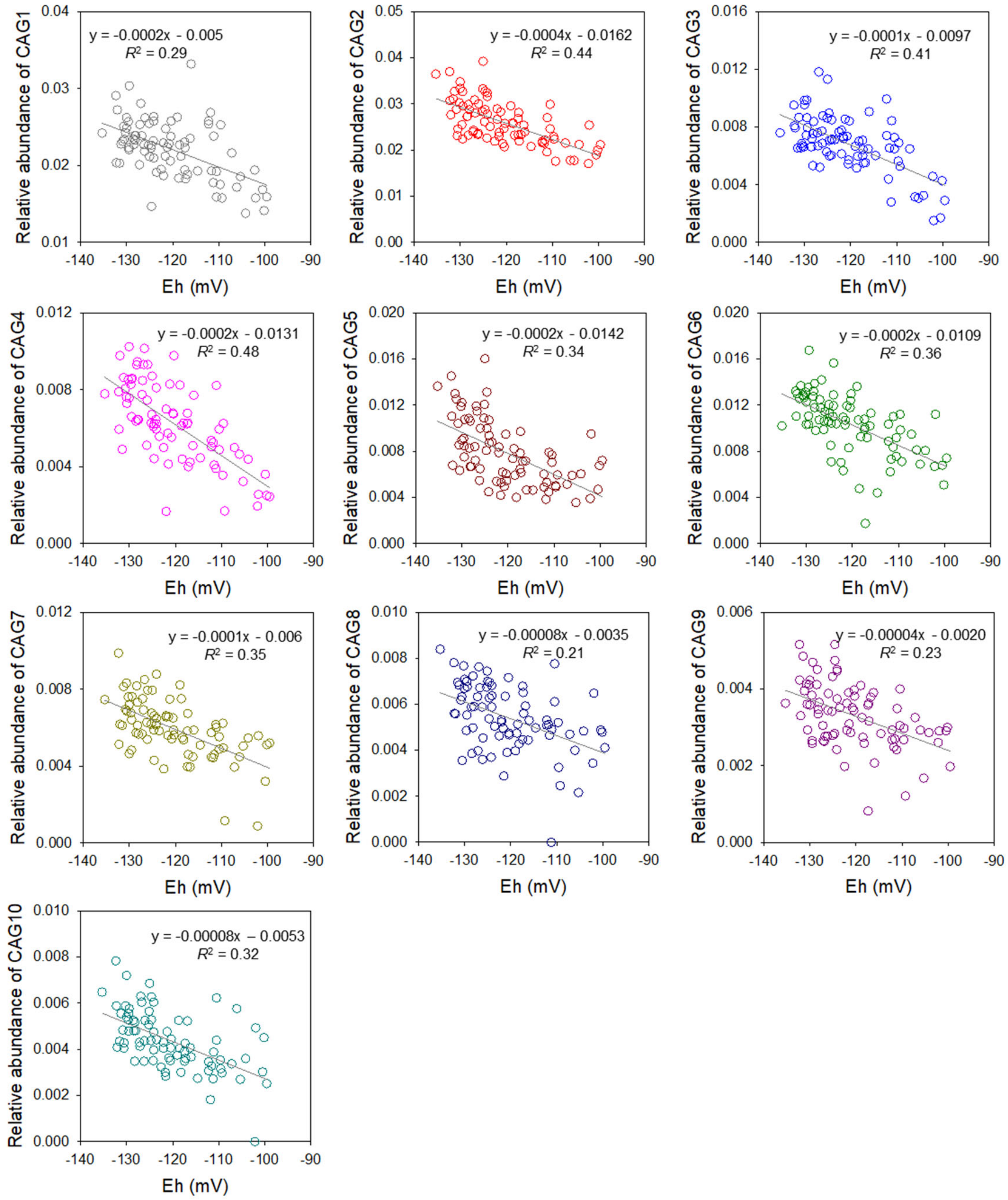

Figure S11. Core ASVs with a positive relationship (r > 0.6, P < 0.05) to husk DMA concentration in TC (A-F) and SY (G-M) fields.

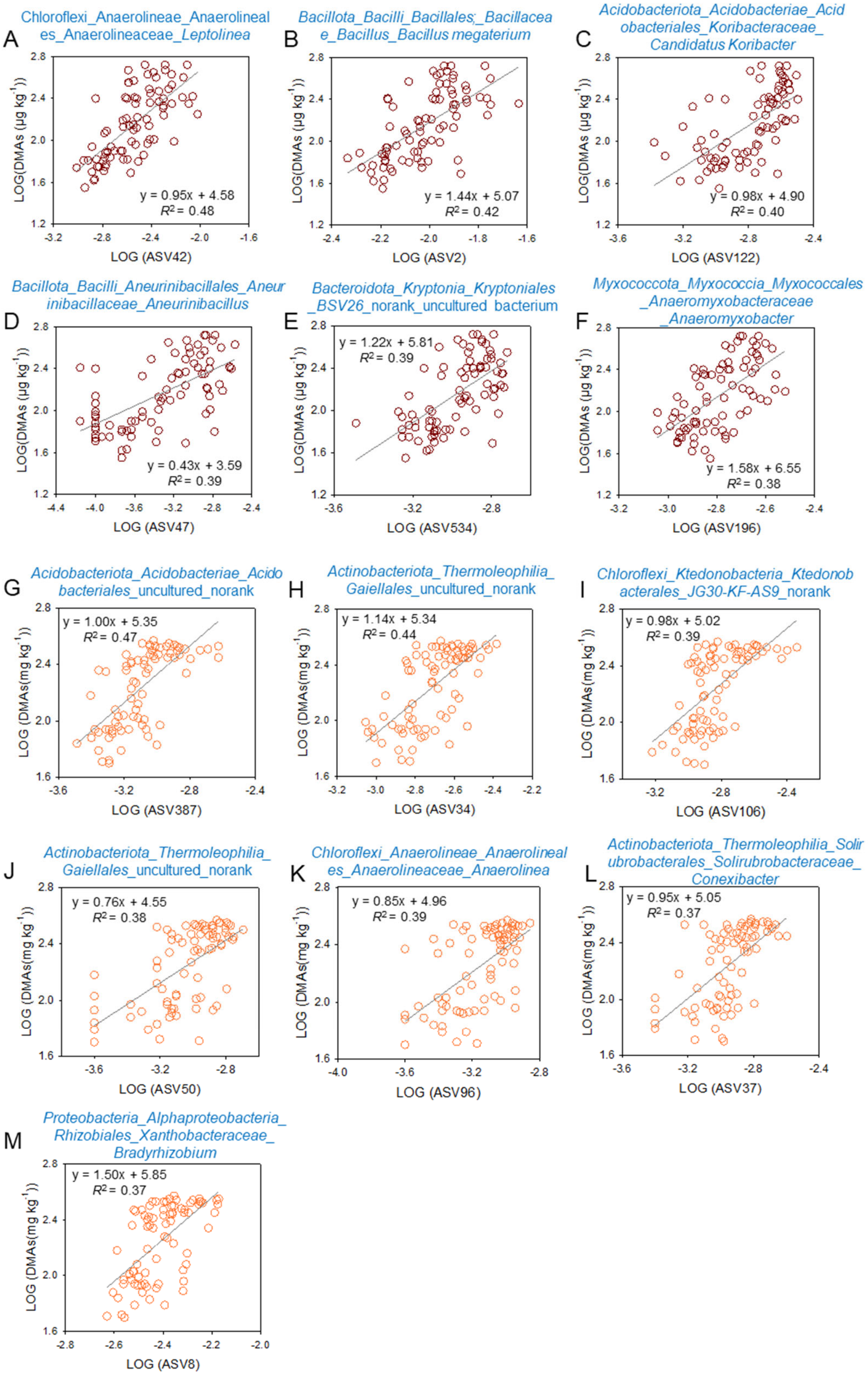

Figure S12. Important ASVs identified by Randomforest for husk DMA concentration in TC (A) and SY (B) fields.

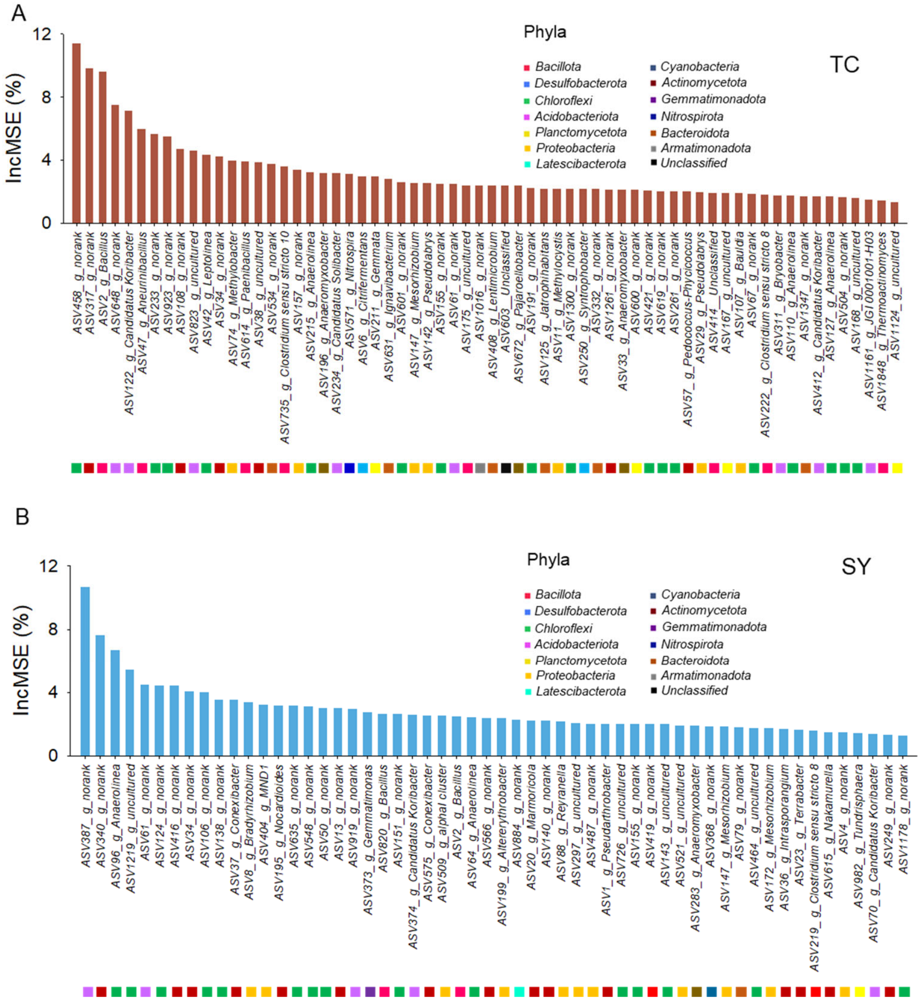

Figure S13. Distribution of *arsM* genes in bacteria and archaea based on metagenomic analysis (A), and distribution of *arsM* genes in different phyla (B).

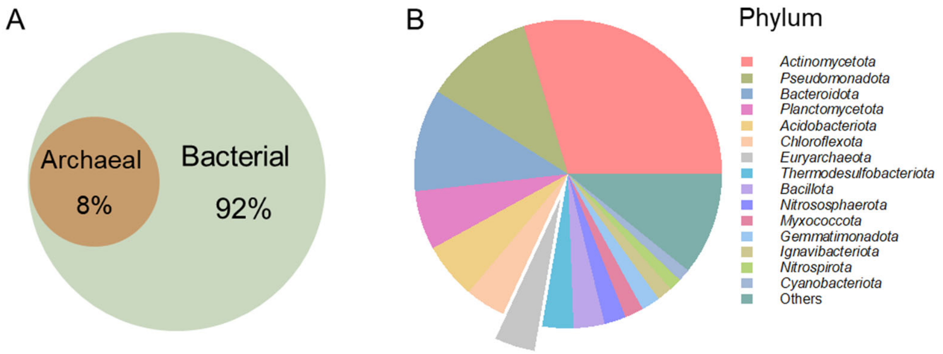

Figure S14. Metagenomic analysis reveals significant differences in the relative abundance of *arsM* genes between the straighthead disease and non-disease patches in TC (A) and SY (B) fields. Number (C) and species (D) of *arsM* genes with a significantly higher abundance in the disease patch and shared between TC and SY fields. Data in A, B and D are means ± SD (n = 3).

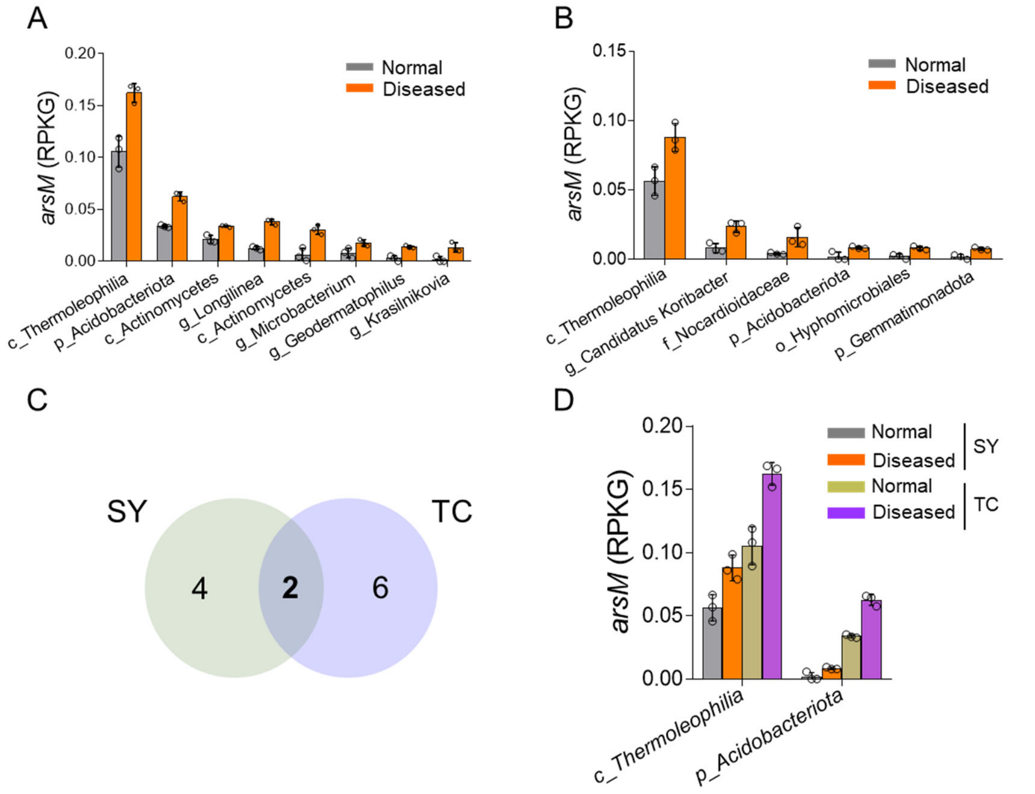

Figure S15. Metagenome-assembled genomes (MAGs) of archaea in TC and SY soils. *arsM* and *mcrA* genes were found to coexist in MAGs via blast against the database. Star and triangle represent the presence of *arsM* and *mcrA* genes in MAGs. Relative abundance of *arsM* gene and transcript in the straighthead disease and non-disease patches are displayed using pie charts. The scale (0.08) indicates sequence divergence.

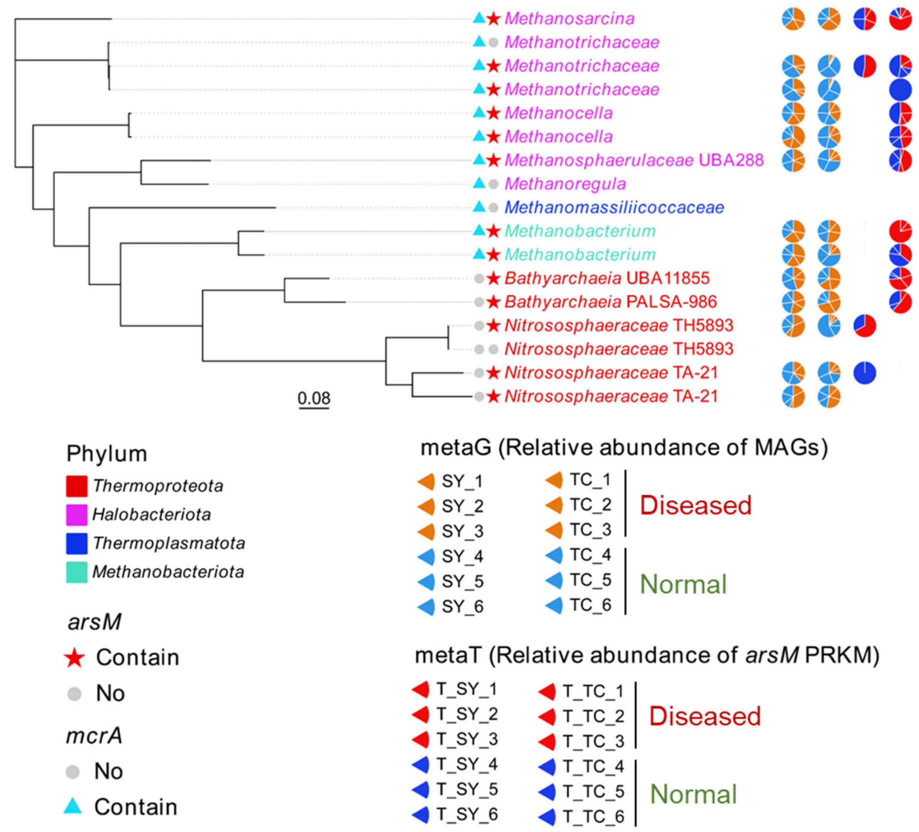

Figure S16. Field slope experiments: spatial variations in soil Eh (A, B), soil total As concentration (C, D), porewater iAs concentration (E, F) and porewater DMA concentration (G, H), and *arsM* gene abundance (I, J) in TC (A, C, E, G, I) and SY (B, D, F, H, J) slope plots.

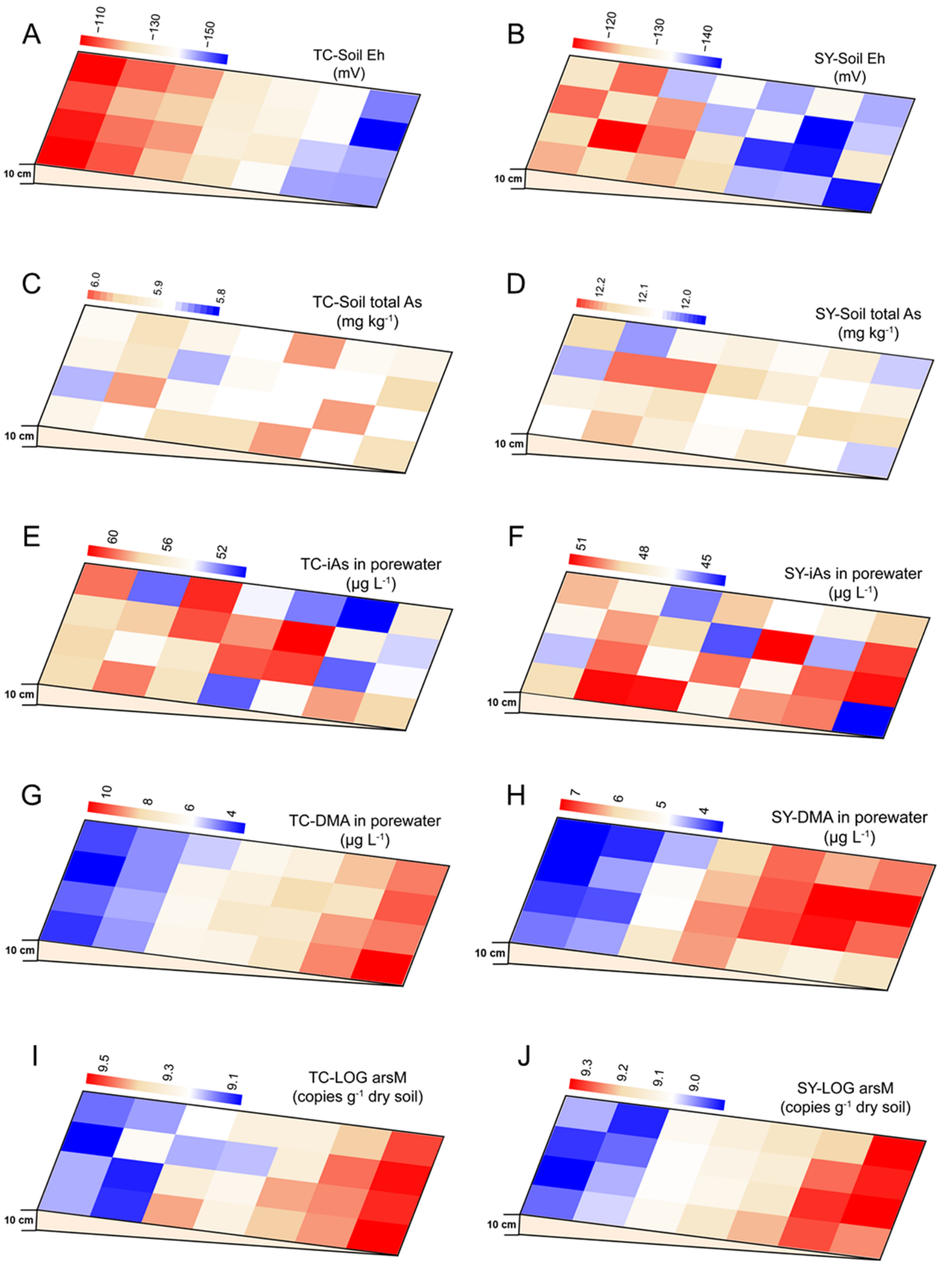

Figure S17. Field slope experiments: spatial variations in husk DMA concentration (A, B), seed setting rate (C, D) and husk iAs concentration (E, F) in TC (A, C, E) and SY (B, D, F) field slopes.

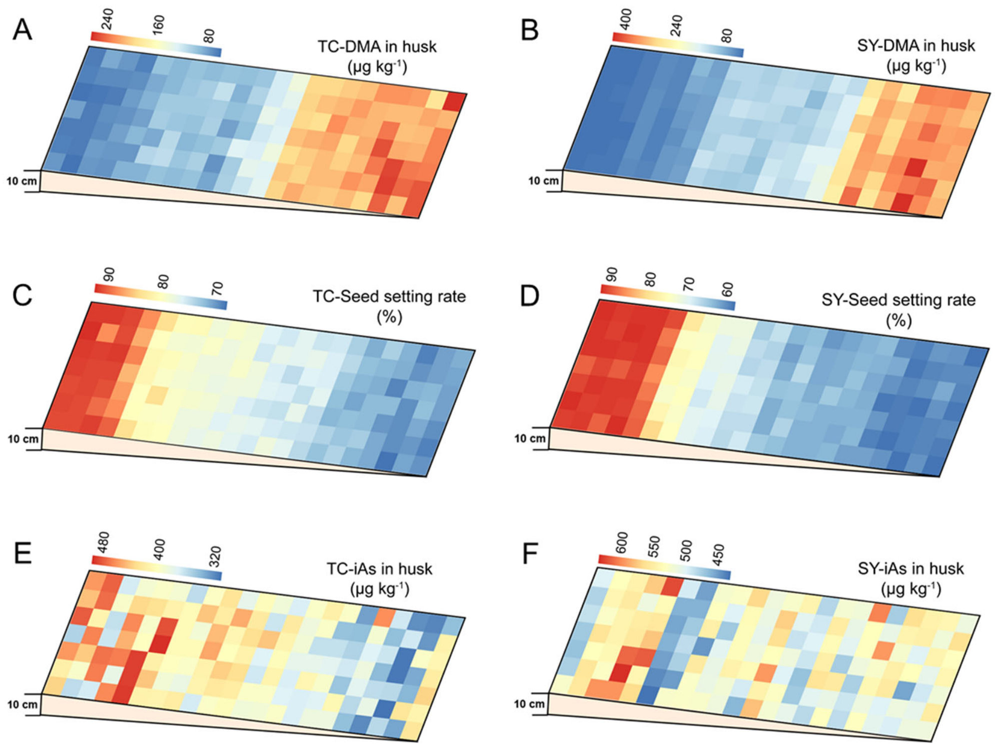

Supplement: Supplementary_Tables_and_Figures_wrae057 [file supplementary_tables_and_figures_wrae057.pdf]
